# State-selective modulation of heterotrimeric Gαs signaling with macrocyclic peptides

**DOI:** 10.1016/j.cell.2022.09.019

**Published:** 2022-09-27

**Authors:** Shizhong A. Dai, Qi Hu, Rong Gao, Emily E. Blythe, Kouki K. Touhara, Hayden Peacock, Ziyang Zhang, Mark von Zastrow, Hiroaki Suga, Kevan M. Shokat

**Affiliations:** 1 Department of Cellular and Molecular Pharmacology, University of California, San Francisco, San Francisco, CA 94158, USA; 2 Howard Hughes Medical Institute, Chevy Chase, MD 20815, USA; 3 Department of Chemistry, Graduate School of Science, The University of Tokyo, 7-3-1 Hongo, Bunkyo-ku, Tokyo 113-0033, Japan; 4 Department of Psychiatry, University of California, San Francisco, San Francisco, CA 94158, USA; 5 Department of Physiology, University of California, San Francisco, San Francisco, CA 94158, USA; 6 Present address: School of Life Sciences, Westlake University, Hangzhou, Zhejiang, China; 7 Present address: Department of Chemistry, University of California, Berkeley, Berkeley, CA, 94720, USA; 8 These authors contributed equally; 9 Lead contact

## Abstract

The G protein-coupled receptor cascade leading to production of the second messenger cAMP is replete with pharmacologically targetable proteins, with the exception of the Gα subunit, Gαs. GTPases remain largely undruggable given the difficulty of displacing high-affinity guanine nucleotides and the lack of other drug binding sites. We explored a chemical library of 10^12^ cyclic peptides to expand the chemical search for inhibitors of this enzyme class. We identified two macrocyclic peptides, GN13 and GD20, that antagonize the active and inactive states of Gαs, respectively. Both macrocyclic peptides fine-tune Gαs activity with high nucleotide-binding-state selectivity and G protein class-specificity. Co-crystal structures reveal that GN13 and GD20 distinguish the conformational differences within the switch II/α3 pocket. Cell-permeable analogs of GN13 and GD20 modulate Gαs/Gβγ signaling in cells through binding to crystallographically defined pockets. The discovery of cyclic peptide inhibitors targeting Gαs provides a path for further development of state-dependent GTPase inhibitors.

## In brief

Cell-permeable Gαs inhibitors regulate G protein signal transduction with high nucleotide-binding-state selectivity and class specificity.

## Graphical Abstract

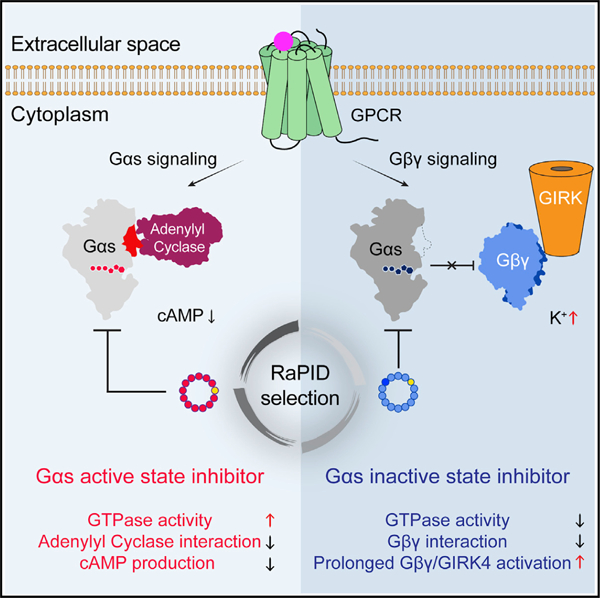

## INTRODUCTION

The family of human GTPases represents a vast but largely untapped source of pharmacological targets. They serve as key molecular switches that control cell growth and proliferation through cycling between tightly regulated ON/OFF states. The role of specific GTPase family members across diverse human diseases has been widely established by cancer genome sequencing (e.g., *KRAS* and *GNAS*) and by familial studies in neurodegenerative disease (e.g., *LRRK2* and *RAB39B*) (Prior et al., 2012; O’Hayre et al., 2013; Alessi and Sammler, 2018; Wilson et al., 2014). Despite the widespread recognition of these disease target relationships, only very recently has the first drug targeting a GTPase K-Ras(G12C) achieved clinical proof of principle (Canon et al., 2019; Hallin et al., 2020) by covalently targeting a somatic mutant cysteine.

Several peptide-based probes that non-covalently target GTPases have been reported, but they either lack proper drug-like properties or have limited target scope (Takasaki et al., 2004; Ja and Roberts, 2004; Johnston et al., 2005; Johnston et al., 2005; Johnston et al., 2006; Ja et al., 2006; Austin et al., 2008). Short linear peptides have been shown to state-selectively target the switch II/α3 pocket in the heterotrimeric G protein α-subunit (Gα). However, linear peptides are not the ideal molecules for drug discovery because of their poor cell permeability and instability in cells.

Cyclic peptides are promising candidates for GTPase drug development. Like linear peptides, cyclic peptides are also capable of targeting protein-protein interfaces (Sohrabi et al., 2020). Peptide cyclization stabilizes the peptide sequence and constrains the peptide conformation for better cell penetration (Dougherty et al., 2019). Cyclic peptide inhibitors of Gα proteins have been reported; for instance, the cyclic depsipeptide natural product YM-254890 targets GDP-bound Gαq with high specificity and potency (Nishimura et al., 2010). Despite the highly conserved structure of G proteins and the recent total synthesis of YM-254890, efforts to use this macrocycle as a scaffold from which to discover inhibitors of other G proteins (Gαs, Gαi) have not been successful (Kaur et al., 2015; Xiong et al., 2016; Zhang et al., 2017), likely due to the limited chemical diversity of available YM-254890 analogs. We therefore reasoned that screening an ultra-large library of cyclic peptides against a given nucleotide binding state of Gαs might allow us to discover Gαs inhibitors that discriminate between the active and inactive states of Gαs and potentially open the remainder of the GTPase family to pharmacological studies.

The Random nonstandard Peptide Integrated Discovery (RaPID) system (Yamagishi et al., 2011) merges the flexibility of an *in vitro* translation system (Murakami et al., 2003, 2006; Ramaswamy et al., 2004; Xiao et al., 2008) with mRNA display, enabling the screening of exceptionally large macrocyclic peptide libraries (>10^12^ molecules) against challenging targets (Passioura and Suga, 2017). Here, we report the discovery by the RaPID system of two macrocyclic peptides, GN13 and GD20, and their analogs cpGN13 and cpGD20, that are cell-permeable, nucleotide-state-selective inhibitors of Gαs, with high selectivity over other G protein subfamilies.

## RESULTS

### Selection of cyclic peptides that bind to the active or inactive state of Gαs

**Figure 1.**
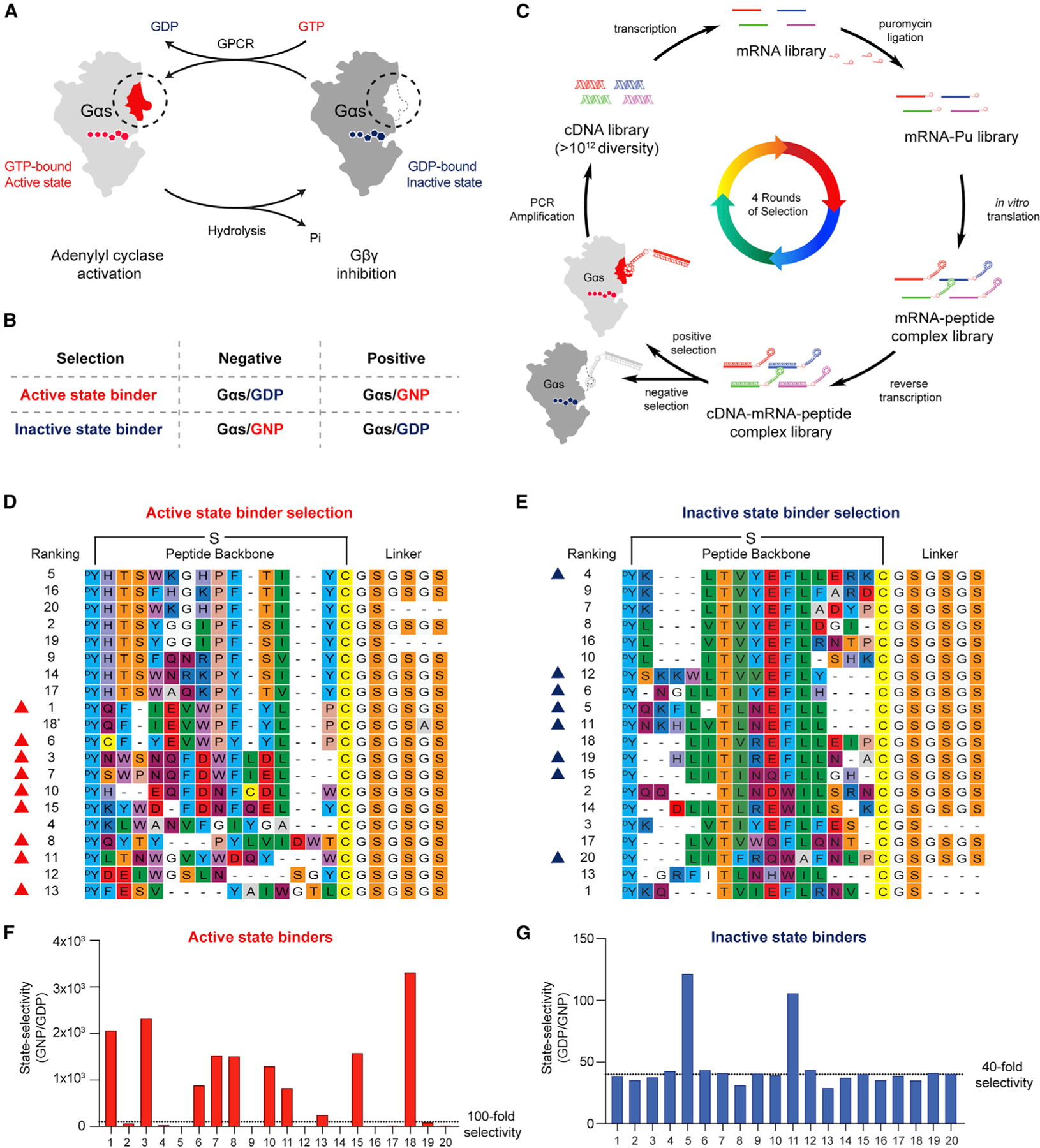
RaPID selection of state-selective Gαs binding cyclic peptides (A) Gαs adopts distinct conformations, governed by its nucleotide binding state. Switch regions are highlighted with a circle. (B) A selection strategy to achieve state-selectivity of Gαs binders. (C) Illustration of the RaPID selection. e.g., Gαs active-state binder selection, positive selection, Gαs/GNP (light gray); negative selection, Gαs/GDP (dark gray). (D and E) Sequence alignment of top 20 cyclic peptides from the R4 pools. The 18^th^ hit (D, asterisk) was not selected because it has the same core sequence as the first peptide. (F and G) State selectivity was determined by comparing peptide-mRNA-cDNA complex binding to GDP- or GNP-bound Gαs. Cyclic peptides with high selectivity are marked with triangles and were selected for solid phase synthesis. See also Figure S1.

The RaPID cyclic peptide discovery platform selects for high-affinity cyclic peptide binders, but hits might bind Gαs anywhere on its surface and so might or might not perturb its function. To increase the probability of selecting function-perturbing hits, we took advantage of the fact that when Gαs switches from the GDP-bound inactive state to the GTP-bound active state, significant conformational changes occur at the switch I, II, and III regions (Lambright et al., 1994), which are known to bind protein partners such as Gβγ or adenylyl cyclases (AC) (Liu et al., 2019; Tesmer et al., 1997) (Figure 1A). We reasoned that performing a positive selection against one state of Gαs and a negative selection against the other state would enrich for binders to the switch regions, and that these binders would be likely to state-selectively disrupt Gαs function.

To select Gαs active-state binders, we performed a positive selection with wild-type (WT) Gαs bound to the non-hydrolyzable GTP analog GppNHp (5ʹ-guanylyl imidodiphosphate [GNP]) and a negative selection against GDP-bound WT Gαs. A parallel Gαs inactive-state binder selection was performed using GDP-bound WT Gαs as the positive selection and GNP-bound WT Gαs as the negative selection (Figure 1B). There are short and long isoforms of Gαs, which are splice variants that differ from each other in the hinge region between the Ras domain and the helical domain (Seifert et al., 1998). If not otherwise noted, the short isoform was used in our study.

Starting from a cDNA library, each round of selection included PCR amplification of the cDNA library, *in vitro* transcription into an mRNA library, ligation with a puromycin linker, and translation to generate a peptide library covalently conjugated to their encoding mRNA library (Figure 1C). The library peptides contain an N-chloroacetyl-D-tyrosine at the N terminus, followed by 8–12 random proteinogenic amino acids encoded by NNK codons (N = G, C, A or U; K = G or U), a cysteine residue, and a GSGSGS linker (G = glycine; S = serine, Figures 1D and 1E). Cyclization occurs spontaneously between the chloroacetyl group and the thiol group of the downstream cysteine residue. The peptide-ligated mRNA library was further reverse-transcribed into a cDNA-mRNA-peptide library, subjected to a negative selection against one state of Gαs, then followed by a positive selection against the other state of Gαs (Figure 1C).

After four rounds of selection (R1–R4), cyclic peptide binders for Gαs/GNP or Gαs/GDP were enriched (Figures S1A and S1B) and identified by next generation sequencing (NGS). The sequences of the top 20 hits are shown in Figures 1D and 1E. Selective cyclic peptides from the R4 pool were characterized by comparison selection against the respective positive and negative protein baits (Figures 1F and 1G, see also Figure S1C). Nine of the top 20 hits from the active-state binder selection (with >100-fold selectivity for Gαs/GNP over Gαs/GDP, red triangles in Figure 1D) and eight of the top 20 hits from the inactive-state binder selection (with >40-fold selectivity for Gαs/GDP over Gαs/GNP, blue triangles in Figure 1E) were chosen for further analysis. To evaluate the cyclic peptide hits without the appended DNA/mRNA duplex, residues from N-chloroacetyl-D-tyrosine to glycine (after the anchor cysteine residue) of the selected peptides were chemically synthesized, followed by cyclization.

### Active-state binding cyclic peptide GN13 blocks Gαs-mediated AC activation

**Figure 2.**
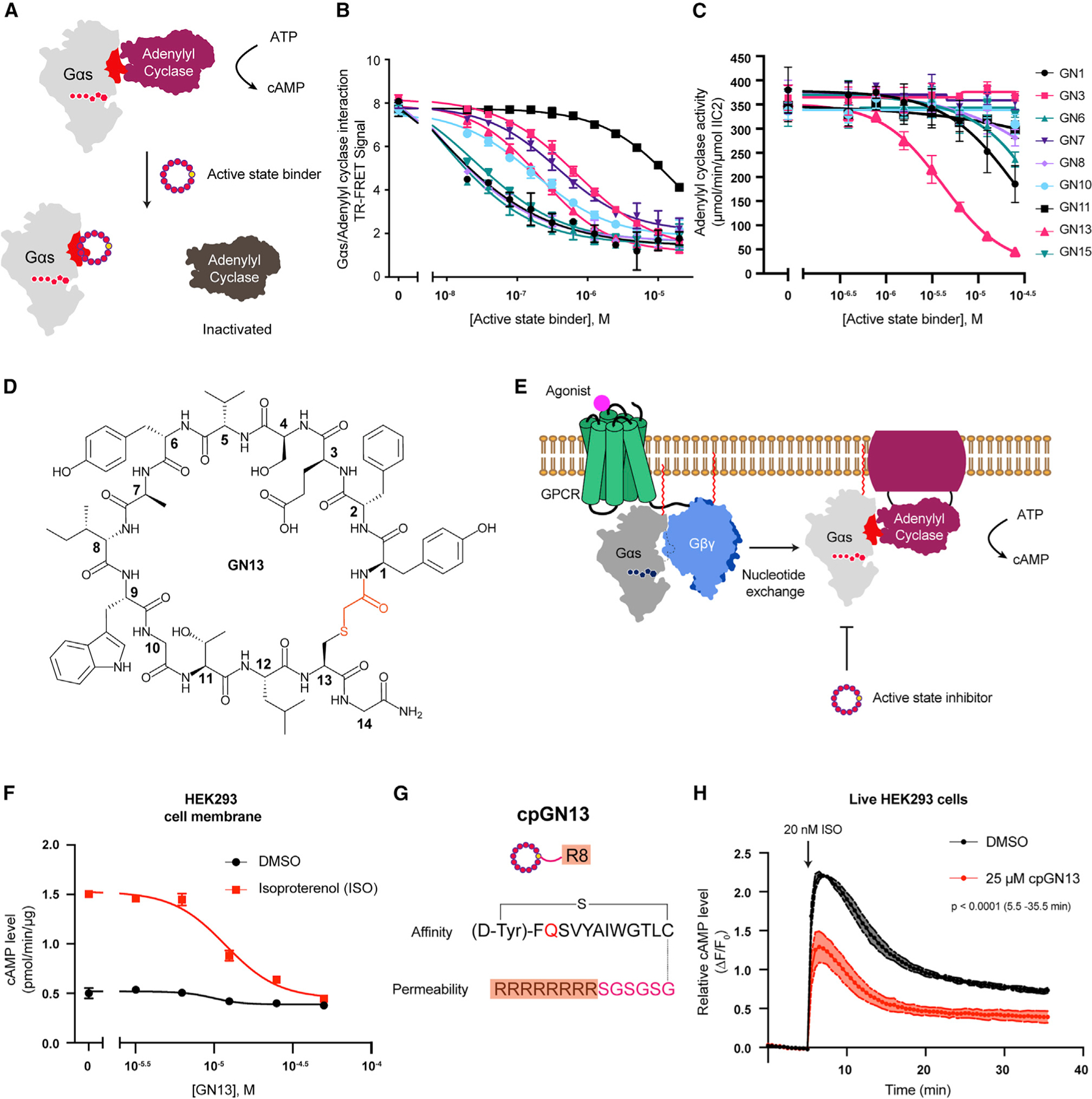
Gαs active-state inhibitor GN13 inhibits Gαs-mediated adenylyl cyclase activation (A) Illustration of active-state binders inhibiting Gαs-mediated AC activation. (B) Active-state binders inhibited PPI between Gαs/GNP and AC. Mean ± SD, n = 3. (C) Gαs/GNP-mediated AC activation was inhibited by active-state binders. Mean ± SE, n = 3. (D) Structure of the resynthesized cyclic peptide GN13. (E) Illustration of GN13 inhibiting GPCR-stimulated Gαs/AC activity in cells. (F) GN13 inhibited ISO-stimulated cAMP production in HEK293 cell membranes. Mean ± SD, n = 3. (G) Design of a cell-permeable GN13 analog, cpGN13. (H) Pretreatment with cpGN13 for 10 min inhibited ISO-stimulated cAMP production in live HEK293 cells. Mean ± SD, n = 3. Two-tailed unpaired t tests (data after 5 min). See also Figure S2.

To determine whether active-state binders inhibit Gαs activity, we assayed the ability of Gαs to activate its effector, AC (Figure 2A). We refer to resynthesized active-state binders with a ‘‘GN’’ (Gαs/GNP) preceding their ranking number. We first tested the interaction between Gαs/GNP and AC in the presence of active-sate binders using a fluorescence resonance energy transfer (FRET) assay (Figure S2A). Eight out of nine GN peptides potently inhibited Gαs/AC interaction (Figure 2B). We then performed a reconstituted AC activity assay to test the ability of GN peptides to inhibit Gαs-mediated AC activation (Figure 2A). GN13 was the most potent among the top hits, with an IC_50_ of 4.15 ± 1.13 μM (Figures 2C and 2D). GN13 did not inhibit Gαs-independent AC activity (Figure S2B), suggesting a Gαs-dependent mechanism of inhibition. We measured the binding of GN13 to immobilized Gαs/GNP using biolayer interferometry (BLI). GN13 binds to Gαs/GNP with a *K*_D_ value of 0.19 ± 0.02 μM (Figure S2C, see also Table S3). By contrast, GN13 showed little to no detectable binding to GDP-bound Gαs (Figure S2D).

We next tested the ability of GN13 to inhibit β2-adrenergic receptor (β2AR)-mediated cAMP production. Membrane anchored GDP-bound Gαs forms a heterotrimer with Gβγ in the resting state. Upon agonist stimulation, β2AR activates Gαs by promoting GDP to GTP exchange (Weis and Kobilka, 2018). We hypothesized that GN13 might capture newly generated GTP-bound Gαs and prevent it from binding AC (Figure 2E). We incubated live HEK293 cells or HEK293 cell membranes with GN13 and measured cAMP accumulation with or without β2AR stimulation by isoproterenol (ISO). Although GN13 showed no inhibition in live HEK293 cells, it inhibited ISO-stimulated cAMP accumulation in cell membranes to a background level, with an IC_50_ of 12.21 ± 2.51 μM (Figures S2E and 2F).

The lack of cell activity of GN13 is presumably limited by its poor cell permeability. We sought to improve its permeability by substituting the negatively charged GN13 with a glutamine residue. We evaluated the cell permeability of GN13-E3Q with a chloroalkane penetration assay (CAPA) (Peraro et al., 2018) (Figure S2F). HeLa cells expressing HaloTag localized to the mitochondrial outer membrane were pulsed with chloroalkane-tagged molecules (ct-molecule), washed, chased with chloroalkane-tagged dye (ct-dye), and analyzed by flow cytometry. A lower ct-dye fluorescent signal indicates competition from a higher cytosolic concentration of ct-molecule. We conjugated a chloroalkane tag at the carboxyl terminus (C-term) of GN13 to make ct-GN13-E3Q (Figure S2G). ct-GN13-E3Q exhibited similar biochemical activity to unmodified GN13 (Figure S2H) and showed measurable cell penetration and mild Gαs inhibition in live cells (Figures S2I and S2J). These results indicated that E3Q mutation and C-term modification of GN13 improved its cell permeability while maintaining Gαs interaction. We further augmented the cellular concentration of GN13-E3Q by adding a polyarginine motif (R8), a widely used cell-penetrating peptide, at the C-term of GN13 (Figure 2G) (Bechara and Sagan, 2013). Cell-permeable (cp)GN13 significantly inhibited ISO-mediated cAMP production in live HEK293 cells (Figure 2H). Our results demonstrated that GN13 and its cp analogs can modulate β2AR agonist-stimulated Gαs activity.

### The crystal structure of GppNHp-bound Gαs in complex with GN13

**Figure 3.**
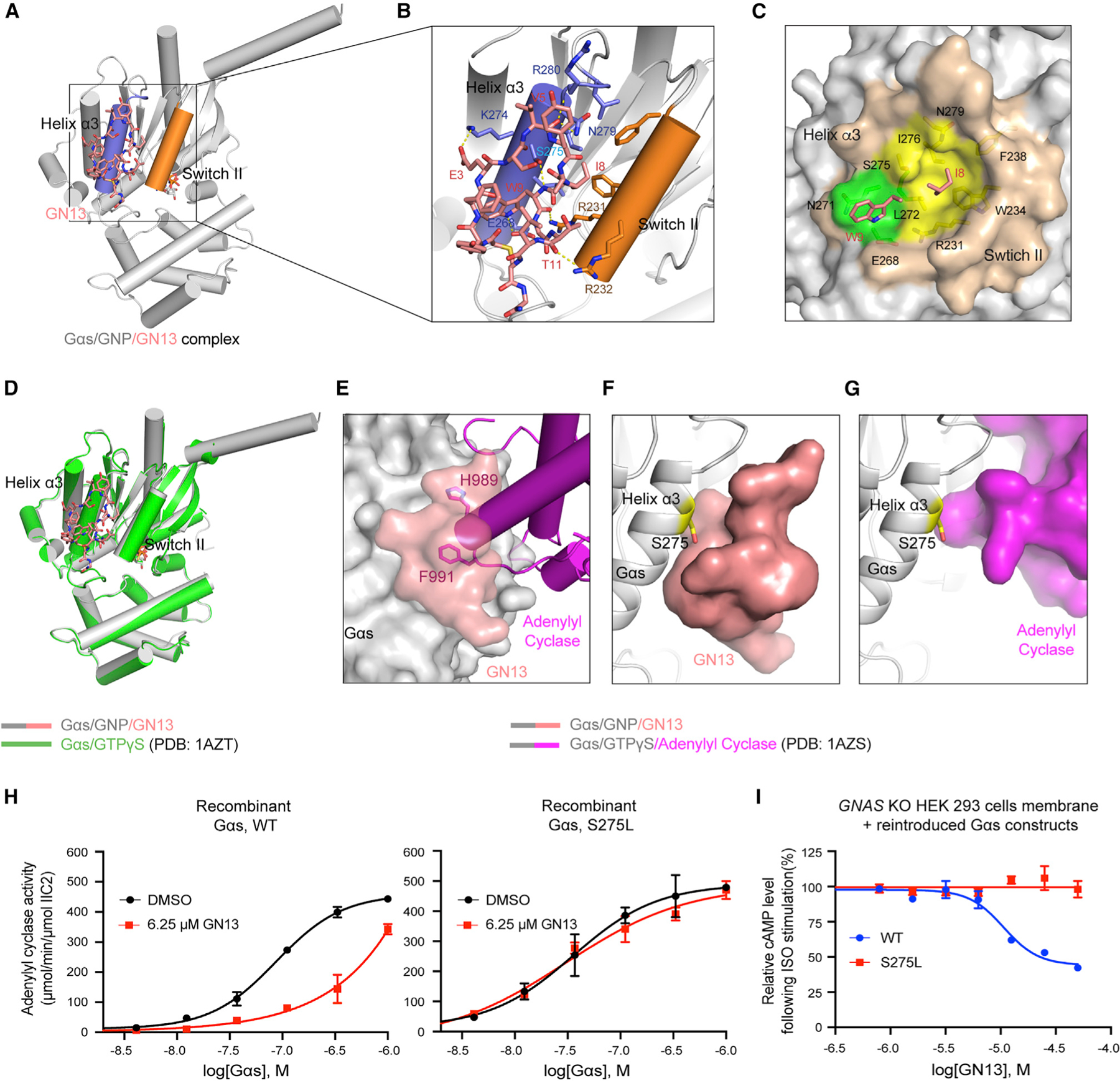
The crystal Structure of GppNHp-bound Gαs in complex with GN13 (A) Overall structure of the Gαs/GNP/GN13 complex. GN13 (salmon) binds in between switch II (orange) and the α3 helix (slate). (B) Structural details of Gαs/GN13 interaction. H-bonds are shown as yellow dashed lines. (C) Close-up view of two Gαs hydrophobic pockets (green and yellow) that accommodate I8 and W9 of GN13 (salmon). Gαs residues that form those pockets are shown as sticks. (D) Alignment of Gαs/GN13 structure (gray) with the structure of Gαs/GTPγS (green, PDB: 1AZT). Root-mean-square deviation = 0.479 Å. (E) Our Gαs/GN13 (gray/salmon) structure was superimposed on the Gαs/AC complex structure (gray/magenta, PDB: 1AZS). GN13 blocks H989/F991 of AC from binding to the same pocket in Gαs. (F and G) Close-up view of the interaction between GN13 (salmon) and the Gαs α3 helix (F) and the interaction between AC (magenta) and the Gαs α3 helix (G, PDB: 1AZS). S275 is shown as sticks. (H) Gαs WT and Gαs S275L have comparable biochemical activities in the AC activation assay (black). GN13 inhibited AC activation by Gαs WT (red, left) but not by Gαs S275L (red, right). Mean ± SD, n = 3. (I) Gαs S275L confers resistance to GN13 inhibition in HEK293 cell membranes. Mean ± SD, n = 3. See also Figure S3 and Table S1.

GN13 inhibited both short and long isoforms of Gαs in the AC activity assay (Figure S3A). To elucidate how GN13 binds to Gαs and inhibits Gαs-mediated AC activation, we solved a co-crystal structure of the GppNHp-bound short isoform Gαs/GN13 complex. The structure was determined by molecular replacement and refined to 1.57 Å (Figure 3A, see also Table S1). GN13 assumes a highly ordered structure through extensive hydrogen bonding networks with three well-defined water molecules (Figures S3B–S3D). One molecule of GN13 binds to the switch II/α3 helix pocket in Gαs through hydrogen bonding and hydrophobic interactions (Figures 3B and 3C). Specifically, the side chain of GN13 accepts a hydrogen bond (H-bond) from Gαs K274; the indole ring of GN13 W9 donates an H-bond to Gαs E268; and the main chains of V5, W9, and T11 in GN13 form H-bonds with Gαs N279, R280, R231, R232, and S275 (Figure 3B). The side chains of GN13 I8 and W9 (IW motif) dock into two Gαs hydrophobic pockets (Figure 3C). To validate these hypothesized interactions, we generated Gαs and GN13 mutants and measured their binding. Although GN13 E3Q mutant retained activity (Figures S2G and S2H), disruption of the H-bond between GN13 × 10^3^ and Gαs K274 with alanine mutations reduced binding by 50% (Figures S3F and S3G). The requirement of a precise Gαs/GN13 H-bond network was confirmed by a series of Gαs mutants (R231A, R232A, E268A, K274A, and N279A) (Figure S3G). Finally, the I8A and W9A mutants of GN13 completely abolished GN13 binding, underscoring the importance of the hydrophobic IW motif (Figure S3F).

Residue W9 in GN13 is centrally located at the interface between GN13 and Gαs (Figures 3B and 3C). In analogous interactions, F991 in AC II (effector of Gαs, Tesmer et al., 1997), W70 in PDEγ (effector of Gαt, Slep et al., 2001), and F108 in Nb35 (Gαs-binding nanobody, Rasmussen et al., 2011) contact the same switch II/α3 clefts of Gαs and Gαt (Figure 3E, see also Figures S3H and S3I). Comparison between the Gαs/GN13 structure and the Gαs/AC complex structure (PDB: 1AZS) suggests that GN13 directly occludes the Gαs/AC interaction, which accounts for the inhibitory effect of GN13 (Figure 3E).

### Structural basis for the nucleotide-state selectivity of GN13

The Gαs/GNP/GN13 structure strongly resembles the Gαs/GTPγS structure (Sunahara et al., 1997), suggesting that GN13 recognizes the active conformation and does not induce significant conformational change upon binding (Figure 3D). GN13 also inhibited oncogenic Gαs mutants (R201C, R201H, R201S, and Q227L) (Figure S3J), which are locked in the active state by catalytic-site mutations (Hu and Shokat, 2018). However, our structure is much less similar to the structure of inactive Gαs/GDP (chain I in PDB: 6EG8, Liu et al., 2019). The N terminus of switch II in Gαs/GDP is unstructured and adjacent to the α3 helix, with nearly half of the GN13/Gαs interface disrupted (Figure S3K). In particular, R232 of switch II in Gαs/GDP is predicted to create a steric clash with GN13 I8, explaining the state selectivity of GN13 for the active state of Gαs.

To assess the cellular specificity of GN13, we designed a GN13-resistant Gαs mutant. We examined the structures of Gαs/GN13 and Gαs/AC and noted that Gαs S275 closely contacts with GN13, but not with AC (Figures 3F and 3G). Mutating S275 to a bulkier residue may create a Gαs mutant that blocks interaction with GN13 but has little effect on AC activation. Indeed, the Gαs S275L mutant maintained a comparable biochemical activity but was resistant to GN13 (Figure 3H, see also Figure S3L). We tested GN13 in the membranes of *GNAS*-knockout (*GNAS*-KO) HEK293 cells that did not express endogenous Gαs protein (Stallaert et al., 2017). GN13 inhibited ISO-mediated cAMP production in *GNAS* KO cell membranes transiently expressing reintroduced WT Gαs, but the inhibitory effect of GN13 was abolished with the Gαs S275L mutation (Figure 3I). These data demonstrate that the observed cellular activity is due to GN13 binding to the switch II/α3 pocket in Gαs.

### Inactive-state binding cyclic peptide GD20 is a Gαs specific guanine nucleotide dissociation inhibitor

**Figure 4.**
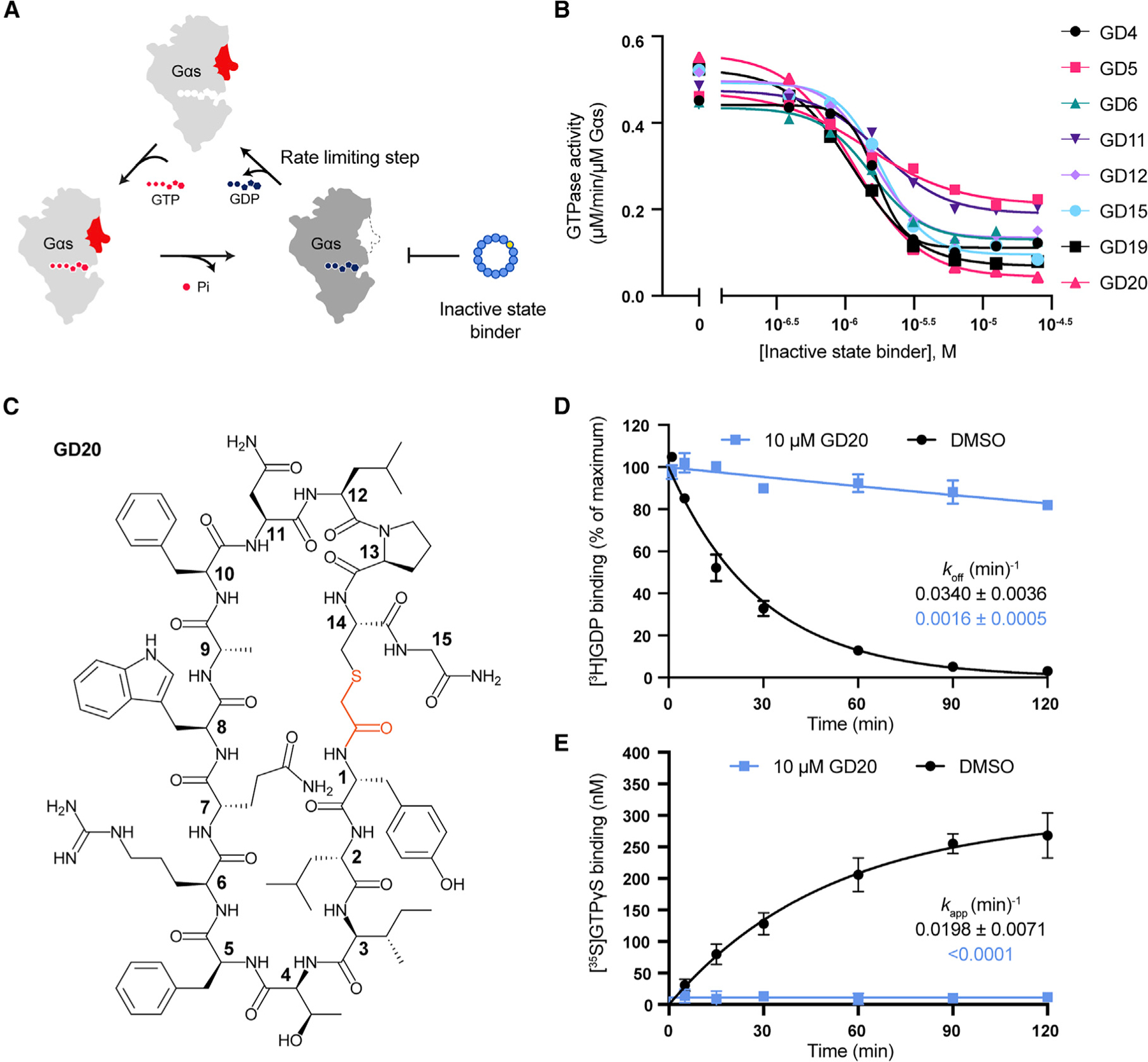
Inactive-state binding cyclic peptide GD20 is a Gαs specific guanine nucleotide dissociation inhibitor (A) Illustration of inactive-state binders inhibiting Gαs steady-state GTPase activity. (B) Gαs steady-state GTPase activity was inhibited by GD peptides. The data represent one measurement. Gαs steady-state GTPase activity in the presence of GD20 was repeated twice in Figure S4A. (C) Structure of the resynthesized cyclic peptide GD20. (D) GD20 inhibited Gαs GDP dissociation. Mean ± SD, n = 3. (E) GD20 inhibited GTPγS binding to Gαs. Mean ± SD, n = 3. See also Figure S4.

Gα GTPase activity hydrolyzes GTP to GDP and rearranges the switch regions to adopt an inactive conformation. This conformation prevents GDP release, which makes GDP dissociation the rate-limiting step of G protein activation (Dror et al., 2015) (Figure 4A, left). To understand how inactive-state binders control Gαs function, we evaluated the steady-state GTPase activity of Gαs in the presence of inactive-state binders (Figure 4B). Resynthesized inactive-state binders are indicated with a ‘‘GD’’ (Gαs/GDP) preceding their ranking number. All of the tested GD peptides strongly inhibited Gαs steady-state GTPase activity. GD20 showed the greatest inhibition, with an IC_50_ of 1.15 ± 0.16 μM (Figures 4B and 4C, see also Figure S4A). GD20 also inhibited the long isoform of Gαs, with an IC_50_ of 1.32 ± 0.17 μM (Figure S4F).

Interestingly, GN13 modestly increased Gαs steady-state GTPase activity (Figure S4A). To understand how GD20 and GN13 regulate Gαs enzymatic activity, we determined rate constants for both GDP dissociation and GTPγS binding. GD20 drastically reduced the GDP dissociation rates (*k*_off_) and the apparent rate of GTPγS binding (*k*_app_), indicating that GD20 is a guanine nucleotide dissociation inhibitor (GDI) (Figures 4D and 4E). On the contrary, GN13 only slightly influenced Gαs GDP dissociation (Figure S4B), and instead slightly increased the maximum GTPγS binding (Figure S4C). The discrepancy between GD20 and GN13 exemplifies how state-selective Gαs binders fine-tune Gαs enzymatic activity. This precise regulation also appears at the G protein family level. Gαi was much less sensitive to GD20 and GN13 (Figures S4D and S4E), highlighting the class-specificity of both cyclic peptides.

### The crystal structure of GDP-bound Gαs in complex with GD20

**Figure 5.**
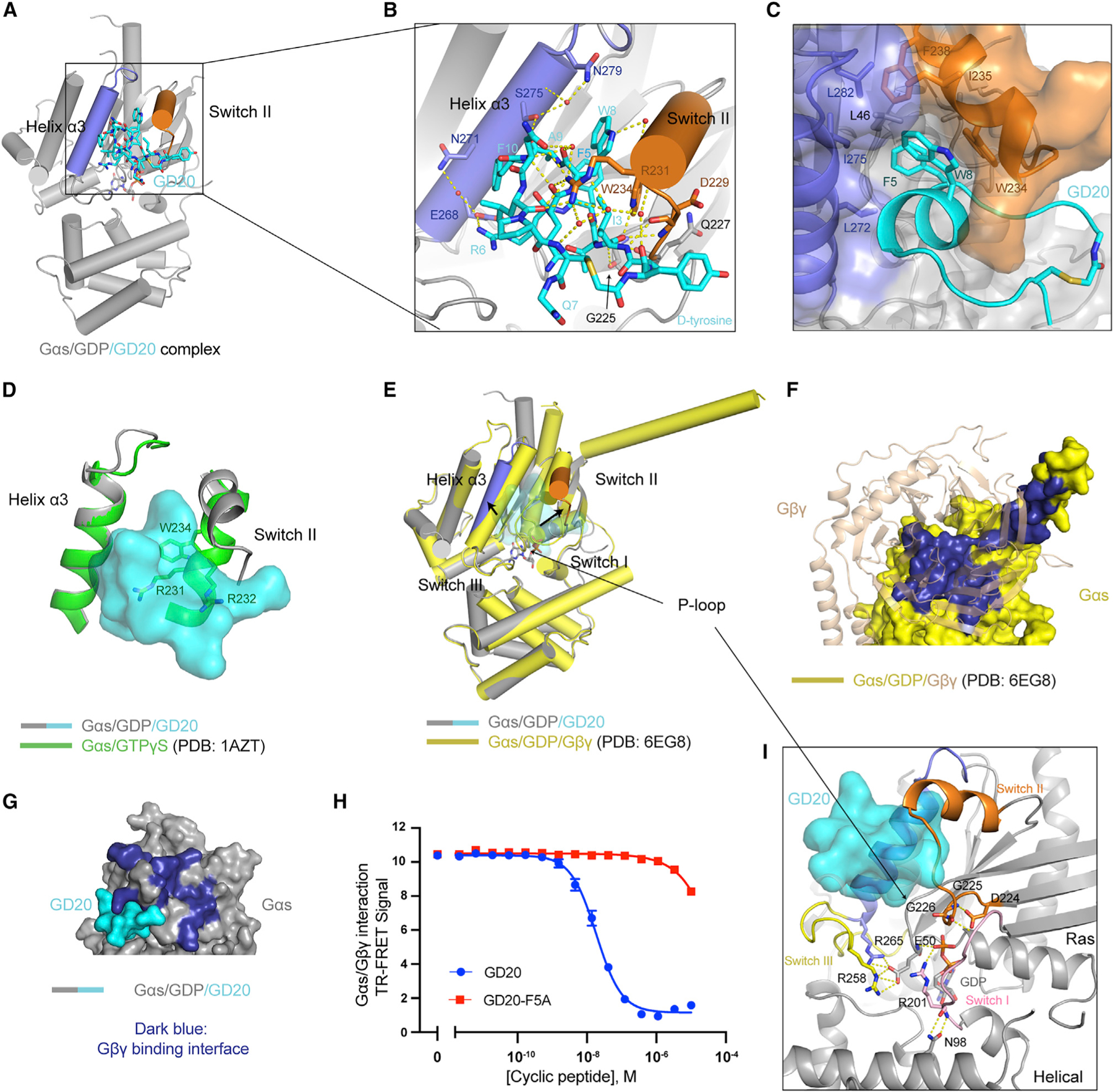
The crystal structure of GDP-bound Gαs in complex with GD20 (A) Overall structure of the Gαs/GDP/GD20 complex. GD20 (cyan) binds in between switch II (orange) and the α3 helix (slate). (B) Structural details of Gαs/GD20 interaction. Ion pair and H-bonds are shown as yellow dashed lines. (C) Close-up view of a hydrophobic pocket in Gαs that accommodates GD20 F5 and W8 (cyan). Gαs residues that form the hydrophobic pocket are shown as sticks. (D) Alignment of Gαs/GD20 complex structure (gray) with the structure of Gαs/GTPγS (green, PDB: 1AZT) in the switch II/α3 pocket. (E) Alignment of Gαs/GD20 complex structure (gray) with the structure of Gαs/GDP (yellow) in the structure of Gαs/Gβ1/γ2 heterotrimer (PDB: 6EG8). Gβγ was hidden for clarity. (F) Structural details of the Gαs (yellow, surface) and Gβγ (wheat, cartoon) binding interface (dark blue) (PDB: 6EG8). (G) The Gβγ binding interface (dark blue) of Gαs is rearranged when GD20 (cyan) binds to Gαs (gray). Gβγ was hidden for clarity. (H) GD20, but not GD20-F5A, inhibited PPI between Gαs/GDP and Gβγ(C68S). Mean ± SD, n = 3. (I) Close-up view of Gαs nucleotide binding pocket in our Gαs/GD20 complex structure. Residues that stabilize GDP binding are shown as sticks. See also Figure S5, Table S2, and Video S1.

To explore how GD20 favors Gαs/GDP and inhibits GDP dissociation, we solved a structure of the Gαs/GDP/GD20 complex. The structure was determined by molecular replacement and refined to 1.95 Å (Figure 5A, see also Table S2). Four well-defined water molecules and a number of intramolecular H-bonds constructed a helical secondary structure in GD20 (Figure S5A–S5E). One molecule of GD20 binds to the switch II/α3 pocket in Gαs through electrostatic interactions, H-bonds, and hydrophobic interactions (Figures 5B and 5C). Specifically, the side chain of GD20 R6 forms a salt bridge with Gαs E268, and this ion pair is stabilized by Gαs N271; the main chain carbonyl oxygen of GD20 A9 forms an H-bond network with Gαs S275 and N279; and the main chain of D229 and the side chain of R231 and W234 in Gαs coordinate a complex H-bond network with I3, W8, N11, L12, C14, and D-tyrosine in GD20 (Figure 5B). These interactions rearrange the flexible Gαs switch II and bury GD20 F5 and W8 inside of a hydrophobic pocket (Figure 5C).

GD20 binds to Gαs/GDP with a *K*_D_ value of 31.4 ± 0.7 nM (Figure S5F, see also Table S3). Single point mutations of GD20, including F5A, R6A, and W8A, nearly completely abolished Gαs binding, confirming the importance of these residues (Figure S5G). The hypothesized interactions were further validated by Gαs mutagenesis studies (Figure S5H): Gαs mutations at contact residues (D229A, R231A, E268A, N271A, and N279A) eliminated GD20 binding to different extents, while mutations at non-contact residues (K274A and R280A) did not influence GD20 binding. The R232A mutation may indirectly reduce GD20 binding through perturbing the switch II conformation.

### Structural basis for the nucleotide-state selectivity and biochemical activity of GD20

GD20 showed high nucleotide-state selectivity for the GDP-bound Gαs (Figure S5I). To understand the mechanism for this selectivity, we superimposed our Gαs/GD20 structure on the structure of active GTPγS-bound Gαs (Sunahara et al., 1997). The rigidified switch II in Gαs/GTPγS—R231, R232, and W234 in particular—clashes with GD20 (Figure 5D). Indeed, GD20 did not inhibit active-state Gαs-mediated AC activation in biochemical or cell membrane experiments (Figures S5J and S5K). Next, we compared our Gαs/GD20 structure with a structure of Gαs/GDP in complex with Gβγ (chain I in PDB: 6EG8, Liu et al., 2019) (Figure 5E). The structural motifs in Gαs (such as switch I, III, and the P loop) that are critical for GDP binding remain unchanged, highlighting the GDP-state selective nature of GD20. However, GD20 binding induces a significant conformational shift at the Gβγ-binding surface by expanding the switch II/α3 pocket. Hence, GD20 may block Gβγ binding to Gαs in a potentially competitive manner (Figures 5F and 5G, see also Video S1). We measured the interaction between Gαs/GDP and Gβγ in the presence of GD20 or a Gαs binding deficient analog, GD20-F5A, using a FRET assay (Figure S5L). Indeed, GD20, but not GD20-F5A, showed potent inhibition of the Gαs/Gβγ interaction, with an IC_50_ of 18.4 ± 2.0 nM (Figure 5H).

The Gαs/GD20 structure also illuminates GD20 GDI activity (Figure 5I). GDP dissociation from Gαs requires conformational changes that weaken GDP affinity and Ras/Helical domain separation to allow GDP release (Dror et al., 2015). GD20 does not engage the GDP exit tunnel and so does not directly occlude GDP release. Instead, GD20 phenocopies the effects of Gβγ GDI activity, stabilizing the conformations of switch I, III, and the P loop in the GDP-bound state. Such a conformational lock not only orients Gαs R201 and E50 to directly capture the β-phosphate of GDP, but also inhibits the spontaneously Ras/Helical domain separation by stabilizing H-bonds between Gαs R201 and N98. As a result, GD20 antagonizes GDP dissociation from Gαs.

### G protein class-specificity of GN13 and GD20

There are four main families of Gα proteins: Gαs, Gαi, Gα12/13, and Gαq. These Gα proteins are structurally similar, yet they transduce divergent G protein-coupled receptor (GPCR) signaling activation by binding to distinct effectors (Syrovatkina et al., 2016). To assess whether GN13 and GD20 can distinguish Gαs from other Gα proteins, we measured their binding to Gαi, Gα13, and Gαq. In contrast to their strong binding to Gαs (Figure S2C and S5F), GN13 and GD20 showed little to no detectable binding to either nucleotide state of Gα13, Gαq, and Gαi at the highest concentration tested (Figure S6A–S6G, and S6J). Furthermore, GD20 disrupted Gα/Gβγ interaction at least 100-fold selectively for Gαs over Gαi (Figure S6K). These results demonstrate that GN13 and GD20 possess excellent G protein class specificity.

**Figure 6.**
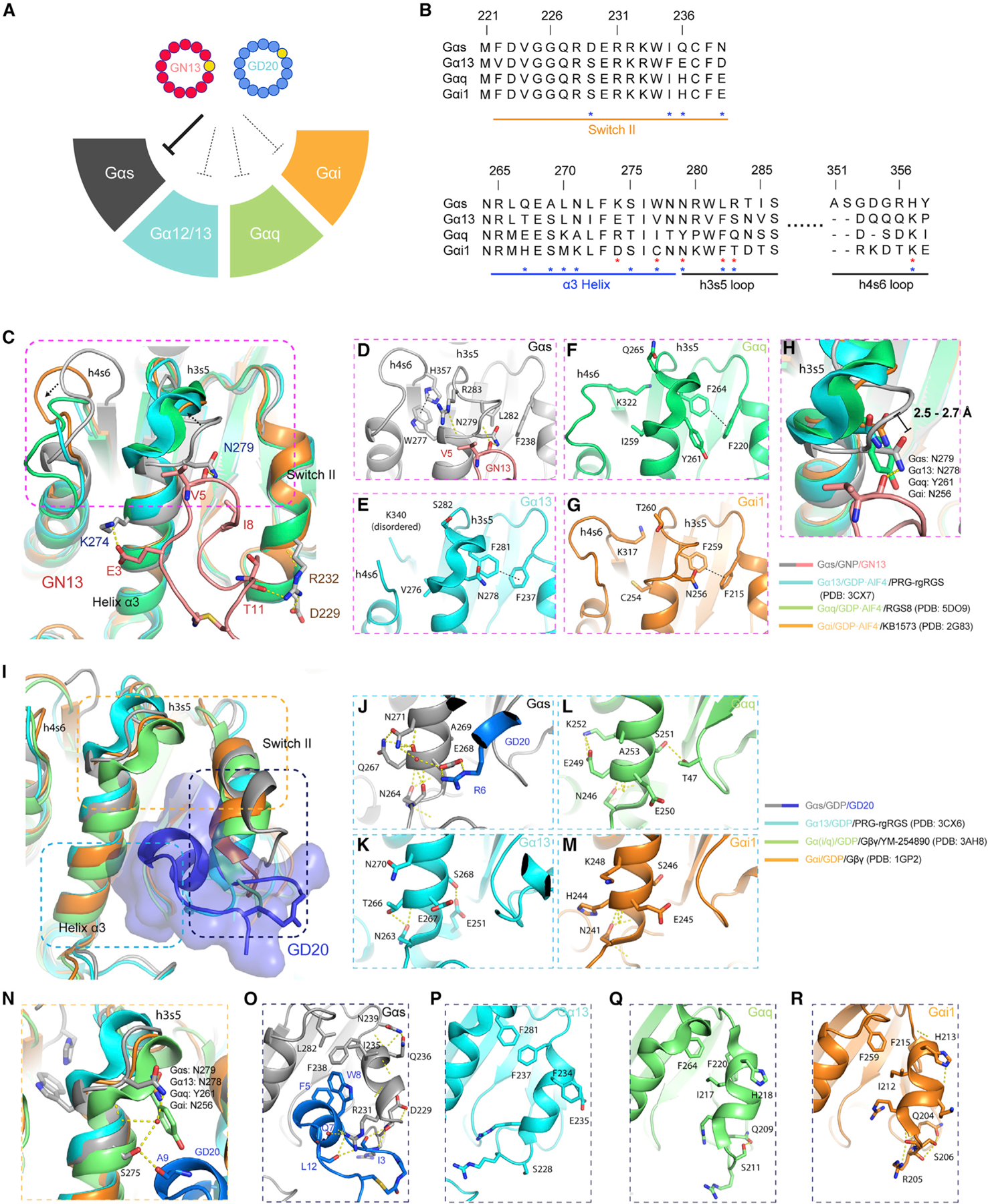
G protein class-specificity of GN13 and GD20 (A) GN13 and GD20 are class-specific Gαs inhibitors. (B) Sequence alignment of Gα proteins around the cyclic peptide binding site. The residue numbering is based on Gαs. Residues that determine the specificity of GN13 (red) or GD20 (blue) are marked with asterisks. (C) The active states of Gα13 (cyan, PDB: 3CX7), Gαq (green, PDB: 5DO9), and Gαi (orange, PDB: 2G83) from their complex structures were superimposed on Gαs/GNP in our Gαs (gray)/GN13 (salmon) structure. (D–G) Structural details of the GN13 binding pocket in different active state Gα proteins. H-bonds are shown as yellow dashed lines. CH/π interactions are shown as black dashed lines. (H) Close-up view of the critical N279 in Gαs/GNP. Homologous residues in other Gα proteins are labeled with different colors. The distances between the Cα of Gαs N279 and the Cα of other homologous residues are indicated. (I) The inactive states of Gα13 (cyan, PDB: 3CX6), Gαq (green, PDB: 3AH8), and Gαi (orange, PDB: 1GP2) from their complex structures were superimposed on Gαs/GDP in our Gαs (dark gray)/GD20 (blue) structure. (J–M) Structural details of the α3 helices in different Gα/GDP proteins. (N) Close-up view of the critical N279 in Gαs/GDP. Homologous residues in other Gα proteins are labeled with different colors. (O–R) Structural details of the switch II regions in different Gα/GDP proteins. See also Figure S6.

To identify G protein specificity determinants of both cyclic peptides, we aligned Gα sequences at the cyclic peptide binding interfaces (Figure 6B) and compared our structures with structures of other Gα proteins in complex with effectors or peptide inhibitors (Chen et al., 2008; Taylor et al., 2016; Johnston et al., 2006; Nishimura et al., 2010; Wall et al., 1995) (Figures 6C–6R).

GDP-AlF_4_^-^-bound active structures of Gα13, Gαq, and Gαi were superimposed on Gαs/GNP in our Gαs/GN13 complex (Figure 6C). There were a few profound differences among Gα proteins. (1) A distinctive π-π stacking between W277 and H357 and a unique R283 in Gαs (the WHR triad) define the positions of the h3s5 and h4s6 loops, which present N279 to form an H-bond with GN13 V5 (Figures 6D and 6H). Changes of the triad in Gα13 (VKS), Gαq (IKQ), and Gαi (CKT) may alter the conformation of loop h3s5 to disrupt interaction with GN13 (Figures 6E–6G). The replacement of N279 by Y261 in Gαq may further limit the Gαq/GN13 interaction (Figure 6H). (2) GN13 I8 docks between F238 and L282 in Gαs. Substitution of L282 with a phenylalanine residue in Gα13, Gαq, and Gαi sterically reshapes this hydrophobic pocket (Figures 6E–6G). The same hydrophobic pocket also controls the binding of other Gα effectors (Chen et al., 2005; Tesmer et al., 2005). (3) Gαs K274 interacts with the negatively charged GN13. Homologous residues in Gα13 (E273) and Gαi (D251) repel the negative charge of GN13. (4) The unique Gαs D229 might participate in an H-bond interaction between GN13 T11 and Gαs R232. However, Gαs D229A mutation did not influence GN13 binding (Figure S3G).

To explain GD20 Gα selectivity, we compared our Gαs/GD20 structure with GDP-bound Gα13, Gαq, and Gαi (Figure 6I). The specificity of GD20 is determined by three major contacts which involve electrostatic interactions, van der Waals interactions, and hydrogen bonding. (1) Gαs N271 positions E268 to interact with GD20 R6 (Figure 6J). Rewired α3 helix H-bond networks in other Gα proteins disfavor this salt bridge formation (Figures 6K–6M). (2) The WHR triad orients N279 in Gαs loop h3s5 for better GD20 binding. N279 and S275 form water-mediated H-bonds with A9 in GD20 (Figure 6N). (3) Switch II has several notable differences between Gα proteins. Three unique residues (D229, Q236, and N239) and a conserved R231 in Gαs support a helical structure in switch II and form H-bond networks with GD20 I3, Q7 and L12 (Figure 6O). Supporting this model, the Gαs D229A and R231A mutants reduced GD20 binding (Figure S5H). The dynamic Gαs switch II also shapes a distinctive hydrophobic pocket (L282-F238-I235) for engagement of GD20. Homologous residues in Gα13, Gαq, and Gαi adopt conformations not compatible with GD20 binding (Figure 6P–6R). In summary, sequence alignment and structural analysis revealed that some of the Gαs residues that directly interact with GN13 and GD20 are not conserved in other Gα proteins, which explains the G protein class specificity of both cyclic peptides.

### A cell-permeable GD20 analog, cpGD20, is a dual-effect G protein modulator

**Figure 7.**
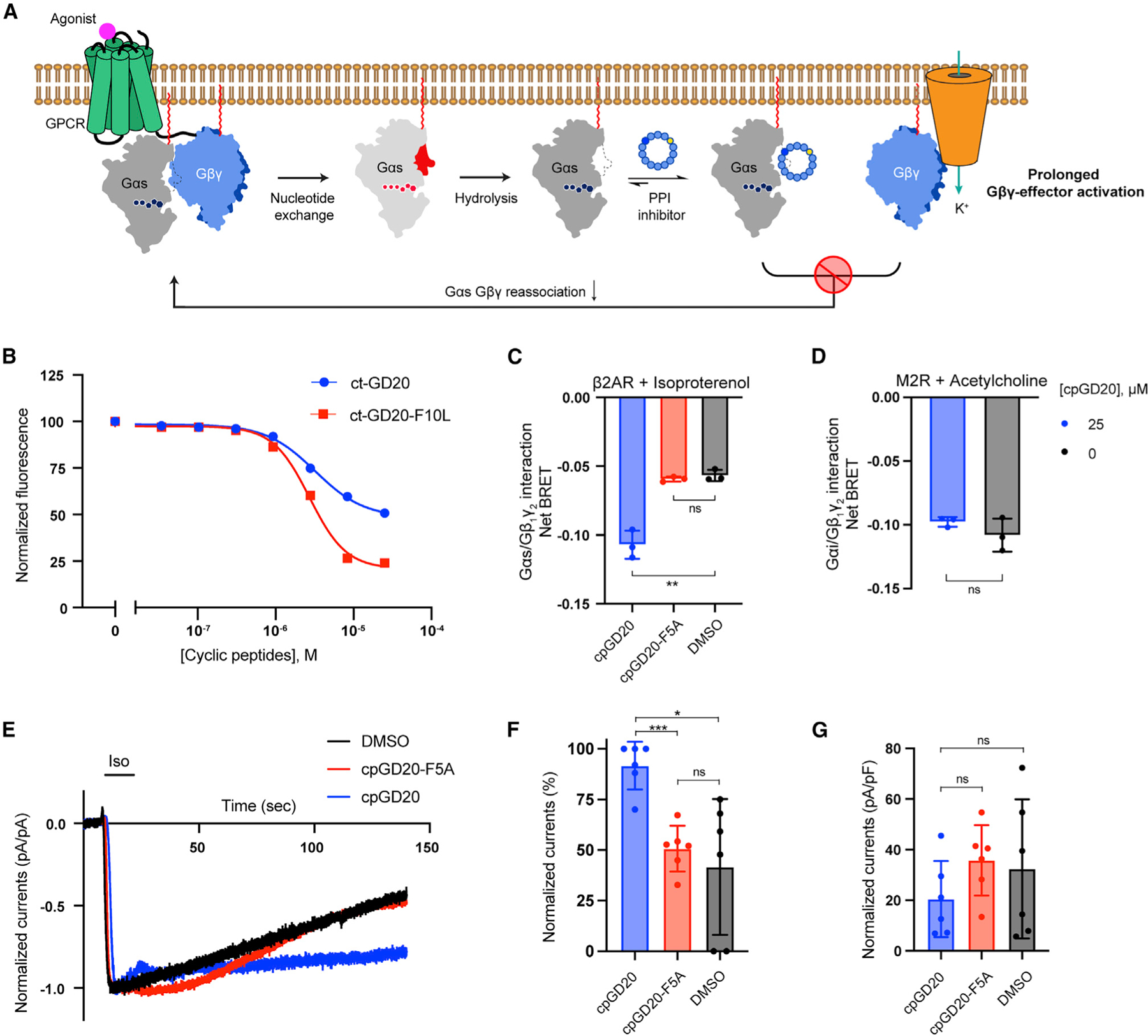
A cell-permeable GD20 analog, cpGD20, is a dual-effect G protein modulator (A) Illustration of Gαs/Gβγ PPI inhibitors acting as dual-effect G protein modulators in cells. (B) CAPA assay results of ct-GD20 and ct-GD20-F10L. Mean ± SD, n = 3. (C) 25 μM cpGD20, but not 25 μM cpGD20-F5A, inhibited Gαs/Gβγ reassociation in HEK293 cells transfected with β2AR and Gαs/Gβ1γ2. Gαs/Gβγ dissociation was measured by BRET signal reduction after 10 nM ISO application. BRET signal was normalized to cells that were not treated with ISO. Mean ± SD, n = 3. Two-tailed unpaired t tests, **p < 0.01, ns p > 0.05. (D) cpGD20 did not inhibit Gαi/Gβγ reassociation in HEK293 cells transfected with M2R and Gαi/Gβ1γ2. Gαi1/Gβγ dissociation was measured by BRET signal reduction after 100 nM ACh application. BRET signal was normalized to cells that were not treated with Ach. Mean ± SD, n = 3. Two-tailed unpaired t tests, ns p > 0.05. (E) Representative voltage-clamp recordings of HEK293 cells transiently transfected with β2AR, GIRK4, Gβγ-Venus, and Gαs. Membrane potential was held at −80 mV. 1 μM ISO was applied as indicated. 25 μM of cpGD20, cpGD20-F5A, or DMSO were added to the pipette solution prior to recordings. (F) The amounts of residual ISO-activated currents after 60 s of washout normalized to the maximum ISO-activated currents. Mean ± SD, n = 6. Two-tailed unpaired t tests with Welch’s correction, *p < 0.05, ***p < 0.001, ns p > 0.05. (G) Maximum ISO-activated currents normalized to the capacitance of the cells. Mean ± SD, n = 6. Two-tailed unpaired t tests with Welch’s correction, *p < 0.05, ns p > 0.05. See also Figure S7.

GPCR signaling releases GTP-bound Gα and free Gβγ to engage their own effectors for downstream signaling. Gα/GDP is a functional ‘‘OFF’’ switch: it tightly reassociates with Gβγ, masking effector-binding surfaces on both Gαs and Gβγ (Gulati et al., 2018). We hypothesized that GD20, a Gαs/Gβγ protein-protein interaction (PPI) inhibitor, may both block Gαs/Gβγ reassociation following receptor stimulation and prolong Gβγ-mediated effector activation (Figure 7A).

We first tested the cell permeability of GD20. The C-terminal residue of GD20 was conjugated with a chloroalkane tag to make ct-GD20 (Figure S7A). Although ct-GD20 is cell permeable, an F10L substitution further improved cell penetration (Figure 7B, see also Figures S7B and S7C). cpGD20 (GD20-F10L) retains binding affinity for Gαs/GDP with a *K*_D_ value of 14.5 ± 0.4 nM (Figure S7D, see also Table S3), and showed biochemical activity, state selectivity, and class specificity comparable to GD20 (Figure S7E, and S6H–S6J). cpGD20 disrupted Gαs/Gβγ interaction with an IC_50_ of 14.0 ± 0.6 nM and exhibited almost 100-fold selectivity over Gαi (Figure S6L).

cpGD20 did not inhibit Gαs-mediated cAMP production in live HEK293 cells, confirming its nucleotide-state-selectivity (Figure S7F). We tested whether cpGD20 could inhibit Gαs/Gβγ interaction in HEK293 cells using a bioluminescence resonance energy transfer (BRET2) assay (Olsen et al., 2020). Rluc8 was inserted into a flexible loop region between the αB-αC helices of Gαs (Gαs-Rluc8) and GFP2 was inserted at the N terminus of Gγ2 (GFP2-Gγ) to capture Gαβγ heterotrimer interaction. A decrease in BRET signal indicates Gαβγ dissociation (Figures S7G and S7H). In cells transiently transfected with β2AR, Gαs-Rluc8, Gβ1, and GFP2-Gγ2, ISO stimulated a reference net BRET response. Pretreatment with cpGD20 induced greater net BRET signal between Gαs and Gβγ (Figure 7C, see also Figure S7I). In comparison, the Gαs binding-deficient cpGD20-F5A failed to induce a larger BRET response (Figure 7C). To assess the specificity of cpGD20 at the G protein level, we tested it against Gαi/Gβγ. HEK293 cells transiently expressing Gαi-coupled muscarinic acetylcholine receptor M2 (M2R), Gαi1-Rluc8, Gβ1, and GFP2-Gγ2 were stimulated with the M2R agonist, acetylcholine (ACh). Pretreatment with cpGD20 did not induce a net BRET signal change (Figure 7D). These data suggest that cpGD20 can specifically capture monomeric Gαs after G protein activation and block Gαs/Gβγ reassociation.

We investigated whether cpGD20 could prolong Gβγ-mediated effector activation after Gαs/Gβγ dissociation. We focused on a well-studied Gβγ effector: The G protein-activated inward rectifier K^+^ (GIRK) channel, which produces inward K^+^ current upon Gβγ binding. Voltage-clamp experiments of HEK293 cells transiently transfected with β2AR, overexpressed Gαs/Gβγ trimer, and GIRK4 showed GIRK activation upon ISO stimulation (Figure 7E, black), consistent with previous findings (Touhara and MacKinnon, 2018). Cyclic peptides treatment did not attenuate the amplitude of GIRK activation (Figures 7E and 7G). However, cpGD20, but not cpGD20-F5A or DMSO, significantly delayed GIRK channel deactivation after ISO washout (Figures 7E and 7F). These results suggest that the Gαs-specific inactive-state inhibitor cpGD20 modulates G protein signaling in two ways, liberating Gαs-bound Gβγ while sequestering GDP-bound Gαs.

## DISCUSSION

GPCRs and G proteins comprise the largest human family of signal transducing proteins. Although ~35% of approved drugs target GPCRs, directly targeting the downstream integrator G proteins has the potential for broader efficacy via blocking convergent pathways shared by multiple GPCRs (Bonacci et al., 2006; Gulati et al., 2018). However, there is a striking absence of drug-like chemical matter that specifically targets the Gα proteins in cells. Cyclic peptides bridge the chemical space between small molecules and biologics and being capable of recognizing shallow effector binding pockets at PPI interfaces while also maintaining favorable pharmacological properties. This is demonstrated here by the development of Gαs selective cyclic peptide inhibitors GN13 and GD20 and their analogs, which specifically recognize the Gαs switch II/α3 pocket. Peptide cyclization and introduction of a non-canonical amino acid (D-tyrosine) give these Gαs inhibitors better cell permeability and chemical stability (Figure 7B, see also Figure S2I, Tables S4 and S5), comparable to small molecule drugs. In contrast to the complex cyclic peptide natural product YM-254890, our Gαs-binding cyclic peptides can be easily derivatized through side-chain substitutions. The high-resolution co-crystal structures of Gαs with our cyclic peptides enable us to program the protein-inhibitor interaction for desired biological effects. This tunability is exemplified by two GD20 analogs, cpGD20 and GD20-F5A, in that a single point substitution drastically changed the biochemical and pharmacological properties of GD20, providing opportunities for further optimization.

Gαs is one of the most frequently mutated G proteins in human cancer. Hotspot mutations in Gαs (Q227 and R201) lock Gαs in a constitutively active conformation (Zachary et al., 1990; Hu and Shokat, 2018). The cyclic peptide GN13 recognized this particular Gαs conformation and inhibited all the tested Gαs oncogenic mutants (Q227L, R201C, R201H, and R201S) in the AC activation assay (Figures S3J and S5J). Pharmacologically targeting the Gαs active state with GN13 has demonstrated the ligandability of oncogenic Gαs and opened up potential to uncover molecular mechanism of tumorigenic Gαs signaling.

Both GN13 and GD20 bind at the evolutionally conserved switch II/α3 pocket. This pocket is generally the site of effector binding, with subtle differences conferred by sequence variability between homologous Gα proteins and by binding of different nucleotides. Our diverse chemical library, along with both positive and negative selection, enabled us to survey the sequence space of cyclic peptides and discover selective binders that capture specific conformations of the Gαs switch II/α3 pocket. The resulting Gαs-cyclic peptide pairings are highly class-specific and state-selective and thus could be useful for developing biosensors that directly detect Gαs/GTP or Gαs/GDP in cells (Maziarz et al., 2020). This molecular recognition is complementary to Gαs nanobody sensors that only capture nucleotide-free Gαs (Manglik et al., 2017).

Pharmacological interrogation of GPCR-mediated signaling events has been largely limited to the receptors. The cell-permeable cyclic peptides cpGN13 and cpGD20 offer an opportunity to directly probe the Gαs/Gβγ trimer at the G protein level and represent additional modes of pharmacological intervention in stimulatory GPCR signaling. The active-state inhibitor, cpGN13 inhibits cAMP production in cells by directly competing with the Gαs effector, AC. This mechanism is distinct from a commonly used Gαs inhibitor, cholera toxin, which catalyzes Gαs ADP-ribosylation and leads to transient Gαs activation and subsequent degradation (Chang and Bourne, 1989). The inactive-state inhibitor cpGD20 sequesters monomeric Gαs/GDP and releases Gβγ from inhibition by Gαs/GDP following receptor stimulation. Therefore, cpGD20 could potentially provide a unique approach to elucidate or even rewire Gαs-coupled receptor signaling by activating Gβγ-dependent pathways. Moreover, rapid Gα/Gβγ reassociation terminates canonical GPCR-dependent G protein signaling within seconds (Ghosh et al., 2017). The slow-dissociating Gαs/cpGD20 interaction (Figure S7D and Table S3) may be able to trap inactive-state Gαs and extend Gβγ-dependent signaling.

Our demonstration of the use of the RaPID cyclic peptide platform through both positive and negative selection steps provides proof of principle for a path to discovering cell-permeable, class-specific and state-selective inhibitors of the remainder of the GTPase family.

### Limitations of the study

Although GN13, GD20, and their analogs are strong Gαs binders, with *K*_D_ values in the nanomolar range, their potencies are compromised in cells. This is likely due to the difficulty of competing tight PPIs on cell membranes and the relatively lower cell penetration of cyclic peptides. Optimizing cyclic peptides with non-canonical residues could potentially improve the potency and cp of cpGN13 and cpGD20 to overcome this limitation. Second, we used Gα13, Gαq, and Gαi1 to test the G protein class specificity of both GN13 and GD20, but we have not performed binding experiments with the entire Gα protein family (e.g., Gαolf, Gα11, G12, and others). It will be of interest in the future to test the specificity of GN13 and GD20 against other Gα proteins. Last, we investigated the cellular activities of cpGN13 and cpGD20 with two GPCRs, β2AR and M2R, and one Gβγ effector, GIRK4. It would be worthwhile to test more Gαs-coupled receptors and Gβγ effectors to further explore the scope of their utility.

## STAR★METHODS

### RESOURCE AVAILABILITY

#### Lead contact

Further information and requests for resources and reagents should be directed to and will be fulfilled by the lead contact, Kevan M. Shokat (kevan.shokat@ucsf.edu).

#### Materials availability

Plasmids generated in this study are available from the lead contact. Compounds generated in this study will be available from the lead contact upon execution of a materials transfer agreement. Cells used in this study cannot be sent because they were made using cell lines from other labs.

### Data and code availability

- Data X-ray Crystallography data have been deposited at PDB and are publicly available as of the date of publication. The accession number for the crystal structure of GNP-bound Gαs in complex with the cyclic peptide inhibitor GN13 reported in this paper is PDB: 7BPH. The accession number for the crystal structure of GDP-bound Gαs in complex with the cyclic peptide inhibitor GD20 reported in this paper is PDB: 7E5E.
- This paper does not report original code.
- Any additional information required to reanalyze the data reported in this paper is available from the lead contact upon request.

### EXPERIMENTAL MODEL AND SUBJECT DETAILS

#### Cell culture

HeLa cells stably expressing the Halo-Tag-GFP-Mito construct were provided by the Kritzer lab (Peraro et al., 2018). HEK293 cells used for cADDis were from ATCC (CRL-1573), and were cultured at 37°C, 5% CO_2_ in DMEM (Thermo Fisher Scientific, Cat#11965118) supplemented with 10% heat-inactivated FBS (HyClone, cat# SH30910.03c). In all other cell-based assays, wildtype HEK293, *GNAS* KO HEK293 were provided by the Inoue lab. Wild-type HEK293, *GNAS* KO HEK293 and HeLa cells were cultured at 37°C, 5% CO_2_ in DMEM (Thermo Fisher Scientific, Cat# 11,995,073) supplemented with 10% heat-inactivated FBS (AxeniaBiologix). All the cells are female in origin.

WT Gαs, all the mutants of Gαs, the C1 domain (residues 442–658, VC1) of human ADCY5 (adenylyl cyclase V) and the C2 domain (residues 871–1082, IIC2) of human ADCY2 (adenylyl cyclase II) were overexpressed in *Escherichia coli* BL21(DE3) cultured in Terrific Broth (TB) Medium. Human GNB1 (Gβ1) and GNG2 (Gγ2) were co-expressed in Sf9 insect cells cultured in Sf-900 III SFM medium at 28°C. Human GNB1 (Gβ1) and GNG2 (Gγ2) were co-expressed in Sf9 insect cells cultured in Sf-900 III SFM medium at 28°C. Human Gα(i/13) was expressed in Sf9 insect cells cultured in Sf-900 III SFM medium at 28°C.

### METHOD DETAILS

#### Protein expression and purification

The following proteins were prepared for the AC assay, the radioactivity assay, and the steady-state GTPase assay:

Standard Gαs protein purification (Hu and Shokat, 2018): The gene of residues 7–380 of the short isoform of human Gαs (GNAS, accession number in PubMed: NP_536351) with a stop codon at its end was cloned into the NdeI/XhoI site of a modified pET15b vector, in which a Drice cleavage site (AspGluValAsp↓Ala) inserted between the thrombin cleavage site and the NdeI site. The resulting WT protein sequence is as follows:

AHMSKTEDQRNEEKAQREANKKIEKQLQKDKQVYRATHRLLLLGAGESGKSTIVKQMRILHVNGFNGDSEKATKVQDIKNNLKEAIE TIVAAMSNLVPPVELANPENQFRVDYILSVMNVPDFDFPPEFYEHAKALWEDEGVRACYERSNEYQLIDCAQYFLDKIDVIKQADYVP SDQDLLRCRVLTSGIFETKFQVDKVNFHMFDVGGQRDERRKWIQCFNDVTAIIFVVASSSYNMVIREDNQTNRLQEALNLFKSIWNN RWLRTISVILFLNKQDLLAEKVLAGKSKIEDYFPEFARYTTPEDATPEPGEDPRVTRAKYFIRDEFLRISTASGDGRHYCYPHFTCAVDT ENIRRVFNDCRDIIQRMHLRQYELL

The plasmid was transformed into *E. coli* BL21(DE3). The transformed cells were grown in TB medium supplemented with 50 μg/mL carbenicillin at 37°C until OD600 reached 0.5, and then cooled to 22°C followed by addition of 40 μM IPTG. After overnight incubation, the cells were harvested by centrifugation, resuspended in lysis buffer (150 mM NaCl, 25 mM Tris 8.0, 1 mM MgCl_2_, protease inhibitor cocktail), and then lysed by a microfluidizer. The cell lysate was centrifuged at 19,000 g for 1 h at 4°C. The supernatant was incubated with TALON Resin at 4°C for 2 h, then the resin was washed by 500 mM NaCl, 25 mM Tris 8.0, 1 mM MgCl_2_ and 5 mM imidazole 8.0. Gαs was eluted by 25 mM Tris 8.0, 1 mM MgCl_2_, 250 mM imidazole 8.0, 10% glycerol and 0.1 mM GDP. After adding 5 mM Dithiothreitol (DTT), the eluate was loaded onto a Source-15Q column. Gαs was eluted by a linear gradient from 100% IEC buffer A (25 mM Tris 8.0, 1 mM MgCl_2_) to 40% IEC Buffer B (25 mM Tris 8.0, 1 M NaCl, 1 mM MgCl_2_). The peak fractions were pooled and supplemented with 5 mM DTT. One-half of peak fractions was mixed with equal volume of GNP exchange buffer (150 mM NaCl, 25 mM HEPES 8.0, 2 mM EDTA, 2 mM GNP, 5 mM DTT) for 2 h, followed by addition of 5 mM MgCl_2_. GNP-bound Gαs and GDP-bound Gαs were concentrated and purified by gel filtration (Superdex 200 increase, 10/30) with SEC buffer (150 mM NaCl, 20 mM HEPES 8.0, 5 mM MgCl_2_ and 1 mM EDTA-Na 8.0). The peak fractions were pooled and concentrated for biochemical assay. All mutants of untagged Gαs (WT, S275L, R201C, R201H, R201S, and Q227L) were expressed and purified with the same protocol. The adenylyl cyclase C2 domain of human ADCY2 (residues 871–1082, IIC2) was also expressed and purified with the same protocol, except that no GDP was added during purification.

Adenylyl cyclase C1 domain purification: Residues D628 and S645 in the C1 domain (residues 443–659) of mouse ADCY5 (adenylyl cyclase V) were mutated to glutamic acid and arginine, respectively, resulting a sequence that is the same as the C1 domain of human ADCY5 (residues 442–658). The gene of this sequence was cloned into the NdeI/XhoI site of a pET29b vector. The transformed *E. coli* BL21(DE3) cells were cultured in TB medium supplemented with 50 μg/mL kanamycin at 37°C until OD600 reached 0.5, and then cooled to 22°C followed by addition of 40 μM IPTG. After incubation at 22°C for 4–5 h, the cells were harvested, lysed. After centrifugation, the supernatant was purified by TALON Resin with the same protocol described above. The eluate was mixed with 5 mM DTT and further purified by gel filtration (Superdex 200 increase, 10/30) with SEC buffer (150 mM NaCl, 20 mM HEPES 8.0, 5 mM MgCl_2_ and 1 mM EDTA-Na 8.0).

Gβγ purification: Human Gβ1 with a hexahistidine tag at its N terminus and human Gγ2(C68S) were cloned into pFastBac Dual expression vector. The plasmid was transformed into DH10Bac competent cells to generate bacmid DNA, which was then used to generate baculoviruses in Sf9 insect cells. Sf9 cells grown in Sf-900 III SFM medium with a density of 1.8×10^6^ cells/mL was infected by the baculoviruses. 48 h later, the cells were harvested by centrifugation, and resuspended in lysis buffer (150 mM NaCl, 25 mM Tris 8.0, 1 mM MgCl_2_) supplemented with protease inhibitor cocktail. The cells were disrupted by a microfluidizer. After centrifugation, the supernatant was purified by TALON Resin and gel filtration (Superdex 200 increase, 10/30) with the same buffers used for Gαs purification.

The following proteins were prepared for the RaPID selection:

The gene encoding residues 7–380 of the short isoform of human Gαs (*GNAS*, accession number in PubMed: NP_536351) with an Avi tag and a TEV cleavage site at its N-terminus was cloned into the multiple cloning site 1 of the pETDuet vector. The resulting protein sequence is as follows:

MGSSHHHHHHSGMSGLNDIFEAQKIEWHESSGENLYFQGMSKTEDQRNEEKAQREANKKIEKQLQKDKQVYRATHRLLLLGAGES GKSTIVKQMRILHVNGFNGDSEKATKVQDIKNNLKEAIETIVAAMSNLVPPVELANPENQFRVDYILSVMNVPDFDFPPEFYEHAKALW EDEGVRACYERSNEYQLIDCAQYFLDKIDVIKQADYVPSDQDLLRCRVLTSGIFETKFQVDKVNFHMFDVGGQRDERRKWIQCFNDV TAIIFVVASSSYNMVIREDNQTNRLQEALNLFKSIWNNRWLRTISVILFLNKQDLLAEKVLAGKSKIEDYFPEFARYTTPEDATPEPGED PRVTRAKYFIRDEFLRISTASGDGRHYCYPHFTCAVDTENIRRVFNDCRDIIQRMHLRQYELL

In the same pETDuet plasmid, the gene encoding BirA (accession number in PubMed: NP_418404.1) was inserted between NdeI and XhoI sites of the multiple cloning site 2. This plasmid was transformed into *E. coli* BL21(DE3). The transformed cells were grown in TB medium supplemented with 50 μg/mL carbenicillin at 37°C until OD600 reached 0.5, and then cooled to 22°C followed by addition of 40 μM IPTG. After overnight incubation, 50 μM biotin was added into the culture for 2 h. The cells were harvested by centrifugation after biotinylation and purified using the standard Gαs protein purification protocol.

The following proteins were prepared for the TR-FRET assay and the bio-layer interferometry assay:

The gene of residues 7–380 of the short isoform of human Gαs (GNAS, accession number in PubMed: NP_536351) with a stop codon at its end was cloned into the NdeI/XhoI site of a modified pET15b vector, in which a Drice cleavage site (AspGluValAsp↓Ala) and an Avi tag were inserted at the N-terminus. The resulting WT Gαs protein sequence after Drice protease cleavage is as follows:

AHMGLNDIFEAQKIEWHESKTEDQRNEEKAQREANKKIEKQLQKDKQVYRATHRLLLLGAGESGKSTIVKQMRILHVNGFNGDSEKA TKVQDIKNNLKEAIETIVAAMSNLVPPVELANPENQFRVDYILSVMNVPDFDFPPEFYEHAKALWEDEGVRACYERSNEYQLIDCAQY FLDKIDVIKQADYVPSDQDLLRCRVLTSGIFETKFQVDKVNFHMFDVGGQRDERRKWIQCFNDVTAIIFVVASSSYNMVIREDNQTNR LQEALNLFKSIWNNRWLRTISVILFLNKQDLLAEKVLAGKSKIEDYFPEFARYTTPEDATPEPGEDPRVTRAKYFIRDEFLRISTASGDG RHYCYPHFTCAVDTENIRRVFNDCRDIIQRMHLRQYELL

The gene of residues 2–354 of human Gαi1 (GNAI1, accession number in PubMed: NP_002060.4) with a stop codon at its end was cloned into the NdeI/XhoI site of a modified pET15b vector, in which a Drice cleavage site (AspGluValAsp↓Ala) and an Avi tag were inserted at the N-terminus. The resulting WT Gαi protein sequence after Drice protease cleavage is as follows:

AHMGLNDIFEAQKIEWHEGCTLSAEDKAAVERSKMIDRNLREDGEKAAREVKLLLLGAGESGKSTIVKQMKIIHEAGYSEEECKQYKA VVYSNTIQSIIAIIRAMGRLKIDFGDSARADDARQLFVLAGAAEEGFMTAELAGVIKRLWKDSGVQACFNRSREYQLNDSAAYYLNDLD RIAQPNYIPTQQDVLRTRVKTTGIVETHFTFKDLHFKMFDVGGQRSERKKWIHCFEGVTAIIFCVALSDYDLVLAEDEEMNRMHESMK LFDSICNNKWFTDTSIILFLNKKDLFEEKIKKSPLTICYPEYAGSNTYEEAAAYIQCQFEDLNKRKDTKEIYTHFTCATDTKNVQFVFDAV TDVIIKNNLKDCGLF

The above-mentioned plasmids were transformed into *E. coli* BL21(DE3), respectively. The transformed cells were grown in TB medium supplemented with 50 μg/mL carbenicillin at 37°C until OD600 reached 0.4, and then cooled to 22°C followed by addition of 100 μM IPTG. After overnight incubation, the cells were harvested by centrifugation, resuspended in lysis buffer (150 mM NaCl, 25 mM Tris 8.0, 1 mM MgCl_2_, protease inhibitor cocktail), and then lysed by a microfluidizer. The cell lysate was centrifuged at 14,000 g for 1 h at 4°C. The supernatant was incubated with TALON resin at 4°C for 1 h, then the resin was washed by 500 mM NaCl, 25 mM Tris 8.0, 1 mM MgCl_2_ and 5 mM imidazole 8.0. G protein was eluted by 25 mM Tris 8.0, 1 mM MgCl_2_, 250 mM imidazole 8.0, 10% glycerol and 0.1 mM GDP. After adding 5 mM Dithiothreitol (DTT), the eluate was incubated with Drice protease at 4°C overnight to remove the hexahistidine tag. Purified BirA (A gift from the Wells lab) and biotin were added at 4°C until LC-MS showed complete biotinylation. G protein was loaded onto a Source-15Q column and eluted by a linear gradient from 100% IEC buffer A (25 mM Tris 8.0, 1 mM MgCl_2_) to 40% IEC Buffer B (25 mM Tris 8.0, 1 M NaCl, 1 mM MgCl_2_). The peak fractions were pooled, nucleotide exchanged, and supplemented with 5 mM DTT and 0.1 mM nucleotide, and then concentrated and purified by gel filtration (Superdex 200 increase, 10/30) with SEC buffer (150 mM NaCl, 20 mM HEPES 8.0, 5 mM MgCl_2_ and 1 mM EDTA-Na 8.0). The peak fractions were pooled and concentrated for biochemical assay. All mutants of Gαs (WT, D229A, R231A, R232A, E268A, N271A, K274A, N279A, and R280A) were expressed and purified with the same protocol.

The following proteins were prepared for the bio-layer interferometry assay (Kreutz et al., 2006):

The gene of residues 1–28 of human Gαi1 (GNAI1, accession number in PubMed: NP_002060.4) and the gene of residues 47–377 of human Gα13 (GNA13, accession number in PubMed: NP_006563.2) with a stop codon at its end was cloned into the pFastBacHTA vector, in which a Drice cleavage site (AspGluValAsp↓Ala) and an Avi tag were inserted at the N-terminus. The resulting protein sequence after Drice protease cleavage is as follows:

AHMGLNDIFEAQKIEWHEMGCTLSAEDKAAVERSKMIDRNLREDGERSARLVKILLLGAGESGKSTFLKQMRIIHGQDFDQRAREEF RPTIYSNVIKGMRVLVDAREKLHIPWGDNSNQQHGDKMMSFDTRAPMAAQGMVETRVFLQYLPAIRALWADSGIQNAYDRRREFQ LGESVKYFLDNLDKLGEPDYIPSQQDILLARRPTKGIHEYDFEIKNVPFKMVDVGGQRSERKRWFECFDSVTSILFLVSSSEFDQVLM EDRLTNRLTESLNIFETIVNNRVFSNVSIILFLNKTDLLEEKVQIVSIKDYFLEFEGDPHCLRDVQKFLVECFRNKRRDQQQKPLYHHFTT AINTENIRLVFRDVKDTILHDNLKQLMLQ

Amplified Avi-Gα(i/13) baculovirus stock was generated using the above-mentioned plasmid in Sf9 insect cells. Cells from 2 L of Sf9 culture were harvested 48 h after infection with 15 mL/L of amplified baculovirus stock, resuspended in 100 mL of Lysis Buffer (20 mM HEPES, pH 8.0, 0.1 mM EDTA, 10 mM 2-mercaptoethanol (βME), 3 mM MgCl_2_, 100 mM NaCl, 50 μM GDP, and protease inhibitor cocktail) and then lysed by microfluidizer. The cell lysate was centrifuged at 19,000 g for 1.5 h at 4°C, after which the supernatants were diluted to a final protein concentration of 5 mg/mL with Buffer A (20 mM HEPES, pH 8.0, 10 mM βME, 1 mM MgCl_2_, 100 mM NaCl, 50 μM GDP, and 12.5 mM imidazole, pH 8.0) and loaded onto TALON resin equilibrated with Buffer A. The resin was washed with 20 vol of Buffer B (Buffer A containing 0.4 M NaCl and 20 mM imidazole, pH 8.0), and the chimera was eluted in 10 fractions of 1 vol of Buffer C (Buffer A containing 150 mM imidazole, pH 8.0). Peak fractions were supplemented with 10% glycerol. The eluate was treated with Drice and 20 μL of BirA at 4°C until LC-MS showed complete biotinylation. (Final [MgCl_2_] = 10mM, [ATP] = 10mM, [Biotin] = 50μM). The eluate was nucleotide exchanged and supplemented with 5 mM DTT and 0.1 mM nucleotide, and then concentrated and purified by gel filtration (Superdex 200 increase, 10/30) with SEC buffer (150 mM NaCl, 20 mM HEPES 8.0, 5 mM MgCl_2_ and 1 mM EDTA-Na 8.0). The peak fractions were pooled and concentrated for biochemical assay.

#### RaPID Selection

Selections were performed with thioether-macrocyclic peptide library against biotinylated Gαs. Thioether-macrocyclic peptide libraries were constructed with N-chloroacetyl-D-tyrosine (ClAc^D^Tyr) as an initiator by using the flexible *in vitro* translation (FIT) system (Goto et al., 2011). The mRNA libraries, ClAc^D^Tyr-tRNA^fMet^_CAU_ were prepared as reported (Yamagishi et al., 2011). The mRNA library corresponding for the thioether-macrocyclic peptide library was designed to have an AUG initiator codon to incorporate N-chloroacetyl-D-tyrosine (ClAc^D^Tyr), followed by 8–12 NNK random codons (N = G, C, A or U; K = G or U) to code random proteinogenic amino acids, and then a fixed downstream UGC codon to assign Cys. After *in vitro* translation, a thioether bond formed spontaneously between the N-terminal ClAc group of the initiator ^D^Tyr residue and the sulfhydryl group of a downstream Cys residue.

In the first round of selection, the initial cyclic peptide library was formed by adding puromycin ligated mRNA library (225 pmol) to a 150 μL scale flexible *in vitro* translation system, in the presence of 30 μM of ClAc^D^Tyr-tRNA^fMet^_CAU_. The translation was performed 37°C for 30 min, followed by an extra incubation at 25°C for 12 min. After an addition of 15 μL of 200 mM EDTA (pH 8.0) solution, the reaction solution was incubated at 37°C for 30 min to facilitate cyclization. Then the library was reversed transcribed by M-MLV reverse transcriptase at 42°C for 1 h and subject to pre-washed Sephadex G-25 columns to remove salts. The desalted solution of peptide-mRNA/cDNA was applied to Gαs (positive selection state)-immobilized Dynabeads M280 streptavidin magnetic beads and rotated at 4°C for 1 h in selection buffer (25 mM HEPES pH 7.5, 150 mM NaCl, 1 mM MgCl_2_ and 0.05% Tween 20) containing 0.5 mM corresponding nucleotide and 0.1% acetylated BSA. Bead amounts were chosen that the final concentration of Gαs protein was 200 nM. This process is referred to as positive selection. The selected peptide-mRNA/cDNAs were isolated from the beads by incubating in 1xPCR reaction buffer heated at 95°C for 5 min. The amount of eluted cDNAs was measured by qPCR. The remaining cDNAs were amplified by PCR, purified and transcribed into mRNAs as a library for the next round of selection.

In the subsequent rounds of selection, ligated mRNA from previous round (7.5 pmol) was added to a 5 μL scale reprogrammed *in vitro* translation system. This was incubated at 37°C for 30 min and at 25°C for 12 min. Then 1 μL of 100 mM EDTA (pH 8.0) was added and incubated at 37°C for 30 min. After reverse transcription and subject to pre-washed Sephadex G-25 columns to remove salts, negative selection was performed by adding the desalted solution of peptide-mRNA/cDNA to Gαs (negative selection state)-immobilized Dynabeads M280 streptavidin magnetic beads and rotated at 4°C for 30 min in selection buffer containing 0.1% acetylated BSA. This process was repeated several times by removing the supernatant to fresh beads immobilized with Gαs (negative selection state). The supernatant from the last negative selection was then added to beads immobilized with the positive selection state of Gαs (final conc. 200nM) and rotated at 4°C for 30 min in selection buffer containing 0.5mM corresponding nucleotide and 0.1% acetylated BSA. As described in the first round of selection, the cDNA was quantified with qPCR, amplified with PCR, transcribed and ligated to puromycin. The subsequent selection was repeated for several rounds until a significant enrichment of cDNA was observed for positive selection state. The recovered cDNA was then identified by next generation sequencing (Miseq, Illumina).

#### Comparison selection

In comparison selection, ligated mRNA (7.5 pmol) from last round selection was added to a 5 μL scale reprogrammed *in vitro* translation system. After translation, cyclization, reverse transcription and pre-washed with Sephadex G-25 columns, the desalted solution of peptide-mRNA/cDNA library was split equally into three fractions, and perform three paralleled selections with the same amount of blank, GDP-bound Gαs-immobilized or GNP-bound Gαs-immobilized Dynabeads M280 streptavidin magnetic beads, individually. For each of the paralleled selections, the beads were rotate at 4°C for 30 min, washed three times with selection buffer. The remaining cDNAs were then eluted from the beads, quantified by qPCR, followed by Miseq sequencing. Finally, identified sequences from each paralleled selection were compared by normalization of Miseq abundance of the sequence with the qPCR reads of the paralleled selection.

#### Bio-layer interferometry (BLI)

BLI experiments were performed using an OctetRED384 instrument from ForteBio. All experiments were performed at 25°C using BLI buffer (10 mM HEPES pH 7.4, 150 mM NaCl, 1mM MgCl_2_, 0.05% Tween 20, 0.1% DMSO, 0.2 mM GNP or GDP). Cyclic peptides or Gα proteins were diluted to a series of concentrations (Final concentrations were indicated in Figures) in BLI buffer plus 10 μM Biotin. Assays were conducted in Greiner 384well, black, flat bottom polypropylene plates containing the protein solutions, BLI buffer plus 10 μM Biotin for dissociation, and serial dilutions of cyclic peptides to be tested.

Biotinylated proteins or cyclic peptides were immobilized on Streptavidin biosensors by dipping sensors into plate wells containing protein solutions at a concentration of 50–150 nM. Protein loading is around 2–3 nm. Cyclic peptide loading is around 0.2–0.3 nm. Sensors loaded with proteins or cyclic peptides were moved and dipped into wells with BLI buffer plus 10 μM Biotin to block unlabeled Streptavidin. Association-dissociation cycles of were started by moving and dipping sensors to cyclic peptides dilutions and BLI buffer plus 10 μM Biotin wells alternatively. Association and dissociation times were indicated in the figure legend.

Raw kinetic data collected were processed with the Data Analysis software provided by the manufacturer using single reference subtraction in which buffer-only reference was subtracted (For GN13 analysis). Because GD20 analogs have a low level of background binding, we used a double reference subtraction (buffer-only reference and non-protein-loading reference) method to calculate their kinetics values. The resulting data were analyzed based on a 1:1 binding model from which *k*_*o*n_ and *k*_*off*_ values were obtained and then *K*_*d*_ values were calculated.

#### Adenylyl cyclase activity assay

Cyclic peptides (4 mM stock in DMSO) were diluted to 4X stocks with a series of concentrations in reaction buffer (1x PBS 7.4, 0.1% BSA). Gαs at a concentration of 8.5 mg/mL (about 190 μM) in 20 mM HEPES 8.0, 150 mM NaCl, 5 mM MgCl_2_, 1 mM EDTA-Na 8.0 was diluted to 0.5 μM in dilution buffer (1x PBS 7.4, 0.1% BSA, 1 mM EDTA-Na 8.0, 2 mM DTT, 0.1mM MgCl_2_) plus 1mM GNP (For the GDP-bound R201 mutants, GDP was used in the nucleotide exchange experiments). After incubation at room temperature for 1 h to allow nucleotide exchange, 2.5 μL of 4x Gαs dilution was mixed with 1 μL MgCl_2_ stock (20 mM MgCl_2_, 1x PBS 7.4, 0.1% BSA) in an OptiPlate-384, White Opaque 384-well Microplate to lock Gαs in GNP-bound state. 2 μL of 5x AC stock (2 μM VC1, 2 nM IIC2, 150 μM FSK, 1x PBS 7.4, 0.1% BSA) was added, followed by addition of 2.5 μL 4X cyclic peptides stock. Reaction mixture was further incubated at room temperature for 2 h and placed on ice for 5 min. cAMP production was initiated by addition of 2 μL of ATP stock (1 mM ATP, 1x PBS 7.4, 0.1% BSA). The reaction was carried out at 30°C for 10 min in a PCR machine and stopped by heating at 95°C for 3 min. The cAMP concentrations were measured by the LANCE Ultra cAMP kit. Final [cyclic peptide]: 0, 0.39, 0.78, 1.56, 3.12, 6.25, 12.5, 25 μM; Final [Gαs]: 125 nM; Final [VC1]: 400 nM; Final [IIC2]: 0.4 nM; Final [FSK]: 30 μM; Final [ATP]: 200 μM. This protocol was used for Figures 2C, 2S2H, S3A, S3J, and S5J.

WT Gαs and S275L mutant at a concentration of 8.5 mg/mL (about 190 μM) in 20 mM HEPES 8.0, 150 mM NaCl, 5 mM MgCl_2_,1 mM EDTA-Na 8.0 were diluted to a series of concentrations in dilution buffer (1x PBS 7.4, 0.1% BSA, 1 mM EDTA-Na 8.0, 2 mM DTT, 0.1mM MgCl_2_) plus 1mM GNP. After incubation at room temperature for 1 h to allow nucleotide exchange, 2.5 μL of 4x each sample was then mixed with 1μL of MgCl_2_ stock (20 mM MgCl_2_, 1x PBS 7.4, 0.1% BSA) in an OptiPlate-384, White Opaque 384-well Microplate. 2 μL of AC/Gβγ 5x stock (2 μM VC1, 2 nM IIC2, 150 μM FSK, 1x PBS 7.4, 0.1% BSA, 10 μM Gβ1/γ2(C68S)) was added, followed by addition of 2.5 μL 25 μM GN13 4x stock in 1x PBS 7.4, 0.1% BSA. Reaction mixture was further incubated at room temperature for 2 h and placed on ice for 5 min. cAMP production was initiated by addition of 2 mL of ATP stock (1 mM ATP, 1x PBS 7.4, 0.1% BSA). The reaction was carried out at 30°C for 10 min in a PCR machine and stopped by heating at 95°C for 3 min. The cAMP concentrations were measured by the LANCE Ultra cAMP kit. Final [cyclic peptide]: 6.25 μM; Final [Gαs]: 0, 1.37, 4.12, 12.3, 37.0, 111, 333,1000 nM; Final [VC1]: 400 nM; Final [IIC2]: 0.4 nM; Final [FSK]: 30 μM; Final [Gβ1/γ2(C68S)]: 2 μM; Final [ATP]: 200 μM. This protocol was used for Figure 3H.

Cell membrane preparation: HEK293cells, *GNAS* KO HEK293 cells were plated two day before transfection at a density of 1M cells per 10cm plate. One plate of *GNAS* KO HEK293 cells was transfected with 4 μg of *GNAS* WT or *GNAS* S275L plasmids. After overnight transfection, cells were lifted with TypLE, washed, resuspended in stimulation buffer (1X PBS, protease inhibitor cocktail, 5 mM MgCl_2_). Cell membranes were disrupted by using the Dounce homogenizer for 25 strokes. Nuclei and unbroken cells were removed by centrifugation for 5 min at 500 g. The supernatant suspension was carefully removed and centrifuged for 30 min at 45K. Cell membranes were suspended in stimulation buffer. The protein concentrations were measured using BCA, and were normalized to 750 μg/mL with stimulation buffer. A final concentration of 0.1% BSA was added into the cell membrane suspension. AC activity assay in cell membranes: 600 μL of cell membrane suspension was mixed with 60 μL of GTP/GDP 20x stock (stock concentration: 10 mM/1 mM). 5.5 μL of the mixture from last step was mixed with 2.5 μL of GN13 4x stocks and incubated at room temperature. After 2 h, membrane/cyclic peptide mixture was transferred on ice for 5 min, followed by the addition of 2 μL of IBMX/ISO/ATP or IBMX/DMSO/ATP 5x stock (5 mM IBMX, 0.2 mM ISO or DMSO, 2.5 mM ATP in stimulation buffer with 0.1% BSA). The reaction was carried out at 30°C for 30 min in a PCR machine and stopped by heating at 95°C for 3 min. The cAMP concentrations were measured by the LANCE Ultra cAMP kit. Final [cyclic peptide]: 0, 0.78, 1.56, 3.12, 6.25, 12.5, 25, 50 μM; Final [membrane]: 375 μg/mL; Final [IBMX]: 1 mM; Final [ISO]: 40 μM; Final [ATP]: 500 μM; Final [GTP]: 500 μM; Final [GDP]: 50 μM. This protocol was used for the HEK293 cell membranes ACassay (Figures 2F, 3I, and S5K).

cAMP concentrations measurement by the LANCE Ultra cAMP kit: A cAMP standard curve was generated in the same plate using the 50 μM cAMP standard in the kit. Before the measurement, the samples were diluted by stimulation buffer (1x PBS 7.4, 0.1% BSA) to 1/60, 1/120, ½40 or 1/480 to make sure the cAMP concentrations were in the dynamic range of the cAMP standard curve. 10 μL of each diluted sample was mixed with 5 μL of 4X Eu-cAMP tracer and 5 μL of 4X ULight-*anti*-cAMP in a white, opaque Optiplate-384 microplate, incubated for 1 h at room temperature, and the time-resolved fluorescence resonance energy transfer (TR-FRET) signals were read on a Spark 20M plate reader. The cAMP standard curve was fitted by the software GraphPad Prism using the following equation in which ‘‘Y’’ is the TR-FRET signal and ‘‘X’’ is the log of cAMP standard concentration (M):

Y=Bottom+Top-Bottom/1+10^LogIC50-X*HillSlope

After obtained the values of the four parameters ‘‘Bottom’’, ‘‘Top’’, ‘‘LogIC50ʹʹ and ‘‘HillSlope’’, we used this equation to convert the TR-FRET signals of the samples into cAMP production values. The cyclic peptides dose dependent inhibition curves were fitted by the following equation to calculate the IC50 of each cyclic peptide:

Y=Bottom+Top-Bottom/1+10^LogIC50-X*HillSlope,

in which ‘‘Y’’ is the cAMP production value, ‘‘X’’ is the log of cyclic peptide concentration (M).

#### Gαs/adenylyl cyclase interaction assay

Cyclic peptides, GN13 and others (4 mM stock in DMSO) were diluted to 5X stocks with a series of concentrations in assay buffer (1X PBS 7.4, 0.1% BSA, 2 mM DTT, 2 mM MgCl_2_). WT Gαs and Gαs S275L mutant at a concentration of 4.6 mg/mL (about 100 μM) in 20 mM HEPES 8.0, 150 mM NaCl, 5 mM MgCl_2_ were diluted to 4 μM in EDTA GNP buffer (1x PBS 7.4, 0.1% BSA, 2 mM EDTA-Na 8.0, 2 mM DTT, 0.1mM MgCl_2_, 1mM GNP). After incubation at room temperature for 1 h to allow nucleotide exchange, Gαs dilutions were mixed with equal volume of MgCl_2_ stock (3.8 mM MgCl_2_, 1x PBS 7.4, 0.1% BSA, 2mM DTT) to lock Gαs in GNP-bound state. GNP-bound Gαs proteins were then diluted to 500 nM (5X stocks) in assay buffer plus 0.5 mM GNP. In an OptiPlate-384 White Opaque 384-well Microplate, 5X Gαs proteins were mixed with 5X GN13 serial dilution stocks, 5X streptavidin XL665 stock (125 nM), 5X AC stock (VC1: 100 nM, IIC2: 200 nM, FSK 0.5mM) and 5X anti-6His-Tb cryptate stock (0.26 μg/mL) in assay buffer for 1 h at room temperature. The plate was read on a TECAN Spark 20 M plate reader using the TR-FRET mode with the following parameters: Lag time: 70 μs, Integration time: 500 μs, Read A: Ex 320(25) nm (filter), Em 610(20) nm (filter), Gain 130, Read B: Ex 320(25) nm (filter), Em 665(8) nm (filter), Gain 165. FRET Signal was calculated as the ratio of [Read B]/[Read A]. In Figure 2A, Final [cyclic peptide]: 0, 0.020, 0.039, 0.078, 0.16, 0.31, 0.62, 1.25, 2.5, 5, 10, 20 μM; Final [Gαs]: 100 nM; Final [VC1]: 20 nM; Final [IIC2]: 40 nM; Final [FSK]: 100 μM. In Figure S3L, Final [cyclic peptide]: 0, 0.677, 2.03, 6.10, 18.3, 54.9, 165, 494, 1481, 4444, 13,333, 40,000 nM; Final [Gαs]: 100 nM; Final [VC1]: 20 nM; Final [IIC2]: 40 nM; Final [FSK]: 100 μM.

#### The cADDis cAMP assay

Real-time cAMP dynamics were measured using the Green Up cADDis cAMP biosensor according to the manufacturer’s protocol. Briefly, cells were lifted using TrypLE Express and resuspended in media supplemented with the appropriate volume of cADDis BacMam. Cells were plated into a 96-well plate at a concentration of 50,000 cells per well and incubated overnight. In the case of 24 h drug pretreatment, cADDis media was replaced with 25 μM drug in DMEM supplemented with 1% dialyzed FBS after 4 h. The next day, plates were washed once with assay buffer (20 mM HEPES pH 7.4, 135 mM NaCl, 5 mM KCl, 0.4 mM MgCl_2_, 1.8 mM CaCl_2_, 5 mM d-glucose) before a 10-min incubation with DMSO or 25 μM drug in a plate reader pre-warmed to 37°C. Fluorescence was detected using an excitation wavelength of 500 nm and an emission wavelength of 530 nm every 30 s. After a 5-min baseline reading, vehicle or 20 nM isoproterenol were added, and fluorescence was measured for 30 min. A baseline fluorescence (F_0_) was calculated for each well by averaging its fluorescence over the 5-min baseline reading, and the fluorescence response at each timepoint was calculated as the change in fluorescence (ΔF=|F - F_0_) normalized to the baseline (F_0_). Each biological replicate represents the average of at least two technical replicates.

#### Steady-state GTPase assay

WT Gαs (both short and long) was diluted to a 6 μM stock (4X) in GTPase assay buffer (20 mM HEPES 7.5, 150 mM NaCl, 1 mM MgCl_2_). The protein was 1:1 (v/v) diluted with 4X cyclic peptide stock (0, 1.56, 3.12, 6.25, 12.5, 25, 50, 100 μM) in GTPase assay buffer, and incubated at 37°C for an hour. The samples were then 1:1 (v/v) diluted with reaction buffer (20 mM HEPES 7.5, 150 mM NaCl, 1 mM MgCl_2_, and 1 mM GTP) and incubated at 37°C. After 30, 50, 70, 90 min, 50 μL of the sample was removed to measure the inorganic phosphate (Pi) concentration by PiColorLock Phosphate Detection kit. A standard curve was made using the 0.1 mM Pi stock in the kit. Final [cyclic peptide]: 0, 0.39, 0.78, 1.56, 3.12, 6.25, 12.5, 25 μM; Final [Gαs]: 1.5 μM; Final [GTP]: 500 μM.

#### GDP dissociation assay

Gα proteins were diluted to 400 nM in the EDTA buffer (20 mM HEPES 7.5, 150 mM NaCl, 1 mM EDTA-Na 8.0, 2 mM DTT). [^3^H]GDP (1 mCi/mL, 25.2 μM) was added to a final concentration of 1.2 μM, followed by cyclic peptides addition. After incubation at 20°C for 30 min, the same volume of assay buffer (20 μM HEPES-Na 7.5, 150 mM NaCl, 2 mM MgCl_2_, and 1 mM GDP) was added to initiate [^3^H]GDP dissociation. Final [cyclic peptide]: 10 μM; Final [Gα]: 187 nM; Final [GDP]: 500 μM. At various points, 10 μL of the sample was removed and mixed with 390 μL of ice-cold wash buffer (20 mM HEPES 7.5, 150 mM NaCl, 20 mM MgCl_2_). The mixture was immediately filtered through a mixed cellulose membrane (25 mm, 0.22 μm) held by a microanalysis filter holder (EMD Millipore). The membrane was washed by ice-cold wash buffer (500 μL x 3), put in a 6-mL plastic vial and air-dried (room temperature 1.5 h). 5 mL of CytoScint-ES Liquid Scintillation Cocktail was added to each vial. After incubation overnight at room temperature, the vial was used for liquid scintillation counting with an LS 6500 Multi-Purpose Scintillation Counter. The GDP dissociation curves were fitted by the software GraphPad Prism using the following equation to calculate the dissociation rates (*k*_*off*_):

$$Y=Y0*\exp\left(-k_{off}*X\right)$$

in which ‘‘Y’’ is the radioactivity (Counts per minute) of the sample at time ‘‘X’’ (minutes), and Y0 is the calculated radioactivity of the sample at the time point 0.

#### GTPγS binding assay

Gα proteins were diluted to 10 μM with dilution buffer (20 mM HEPES 7.5, 150 mM NaCl, 1 mM MgCl_2_, 2 mM DTT, and 20 μM GDP) and incubated with 5X stocks of cyclic peptides at room temperature for 2 h. GTPγS binding was initiated by mixing with the reaction buffer at room temperature (50 nM [^35^S]GTPγS and 100 μM GTPγS in dilution buffer) at room temperature. Final [cyclic peptide]: 10 μM; Final [Gα]: 2 μM; Final [GTPγS]: 100 μM. At various time points, 10 μL of the sample was removed and mixed with 390 μL of ice-cold wash buffer (20 mM HEPES 7.5, 150 mM NaCl, 20 mM MgCl_2_). The mixture was filtered through a mixed cellulose membrane (25 mm, 0.22 μm). The membrane was washed by ice-cold wash buffer (500 μL x 3), put in a 6-mL plastic vial and air-dried (room temperature 1.5 h). 5 mL of CytoScint-ES Liquid Scintillation Cocktail (MP Biomedicals) was added to each vial. After incubation overnight at room temperature, the vial was used for liquid scintillation counting with an LS 6500 Multi-Purpose Scintillation Counter. A standard curve was generated using [^35^S]GTPγS. The radioactive activity (Counts per minute) of the samples were converted to the GTPγS concentration. The GTPγS binding curves were fitted by the software GraphPad Prism using the following equation to calculate the apparent GTPγS binding rates (*k*_*app*_):

$$Y=Plateau*\left(1-\exp\left(-k_{app}*X\right)\right)$$

in which ‘‘Y’’ is the concentration of GTPγS that bound to Gα protein at time ‘‘X’’ (minutes).

#### FRET based Gα/Gβγ interaction assay

Biotinylated avi-Gαs (6-end, WT) and avi-Gαi (FL, WT) were diluted to 32 nM (8X) using assay buffer (1X PBS 7.4, 2 mM DTT, 0.1% BSA, 2 mM MgCl_2_, 0.05% Tween plus 0.5 mM GDP), followed by mixing with a same volume of 8X streptavidin XL665 stock (32 nM in the assay buffer). 8X His-Gβ/γ (C68S) stock (16 nM) and 8X anti-6His-Tb cryptate stock (0.4 μg/mL) were added into the Gα/XL665 mixtures. Finally, 2X stocks of cyclic peptides were (Final cyclic peptide concentrations were indicated in Figures) added with the protein mixtures. After incubation at room temperature for 2 h at room temperature. The plate was read on a TECAN Spark 20 M plate reader using the TR-FRET mode with the following parameters: Lag time: 70 μs, Integration time: 500 μs, Read A: Ex 320(25) nm (filter), Em 610(20) nm (filter), Gain 130, Read B: Ex 320(25) nm (filter), Em 665(8) nm (filter), Gain 165. FRET Signal was calculated as the ratio of [Read B]/[Read A]. Final [cyclic peptide]: 0, 0.002, 0.006, 0.019, 0.056, 0.169, 0.508, 1.524, 4.57, 12.7, 41.2, 123, 370, 1111, 3333, 10,000 nM; Final [Gα]: 4 nM; Final [Gβ1/γ2(C68S)]: 2 nM.

#### Crystallization

GN13/GNP/Gαs complex: Wild type Gαs (residues 7–380) that was preloaded with GNP and purified by gel filtration was concentrated to 10 mg/mL. The protein was then mixed with 1 mM of GNP (50 mM stock in H_2_O) and 0.42 mM of the cyclic peptide GN13 (14 mM stock in DMSO). For crystallization, 0.2 μL of the protein sample was mixed with 0.2 μL of the well buffer containing 0.1 M HEPES 7.2, 20% PEG4000, 10% v/v 2-propanol. Crystals were grown at 20°C in a 96-well plate using the hanging-drop vapor-diffusion method, transferred to a cryoprotectant solution (0.1 M HEPES 7.2, 20% PEG4000, 10% v/v 2-propanol, 150 mM NaCl, 20 mM HEPES 8.0, 5 mM MgCl_2_, 1 mM GNP, 25% v/v glycerol), and flash-frozen in liquid nitrogen.

GD20/GDP/Gαs complex: Wild type Gαs (NCBI Reference Sequence: NP_536351.1, residues 35–380) was preloaded with GDP, purified by gel filtration and then concentrated to 11.6 mg/mL. Before crystallization, the protein was mixed with 5 mM of Dithiothreitol (0.5 M stock in H_2_O), 1 mM of GDP (50 mM stock in H_2_O) and 0.76 mM of the cyclic peptide GD20 (42.6 mM stock in DMSO). For crystallization, 1.5 μL of the protein sample was mixed with 1.5 μL of the well buffer containing 0.1 M Tris 8.2, 26% PEG4000, 0.8 M LiCl. Crystals were grown at 20°C in a 15-well plate using the hanging-drop vapor-diffusion method, and flash-frozen in liquid nitrogen.

#### Data collection and structure determination

The dataset was collected at the Advanced Light Source beamline 8.2.1 with X-ray at a wavelength of 0.999965 Å. Then the dataset was integrated using the HKL2000 package (Otwinowski and Minor, 1997), scaled with Scala (Evans, 2006) and solved by molecular replacement using Phaser (McCoy et al., 2007) in CCP4 software suite (Winn et al., 2011). The crystal structure of GDP-bound human Gαs R201C/C237 mutant (PDB code: 6AU6) was used as the initial model. The structure was manually refined with Coot (Emsley et al., 2010) and PHENIX (Adams et al., 2010). Data collection and refinement statistics are shown in Tables S1 and S2.

#### Chloroalkane penetration assay (CAPA)

The cell lines used for CAPA were HeLa cell lines, generated by Chenoweth and co-workers, that stably express HaloTag exclusively in the cytosol (Peraro et al., 2018). Cells were seeded in a 96-well plate the day before the experiment at a density of 4 × 10^4^ cells per well. The day of the experiment the media was aspirated, and 100 μL of cyclic peptide dilutions in DMEM were added to the cells. Plate was incubated for 19.5 h at 37°C with 5% CO_2_. The contents of the wells were aspirated off, and wells were washed using fresh Opti-MEM for 15 min. The wash was aspirated off, and the cells were chased using 5 μM ct-TAMRA for 15 min, except for the No-ct-TAMRA control wells, which were incubated with Opti-MEM alone. The contents of the wells were aspirated and washed with fresh Opti-MEM for 30 min. After aspiration, cells were rinsed once with PBS (PBS). The cells were then trypsinized, quenched with DMEM, resuspended in PBS, and analyzed using a benchtop flow cytometer (CytoFLEX, Beckman). Final [cyclic peptide]: 0, 0.034, 0.10, 0.31, 0.93, 2.78, 8.33, 25 μM.

#### BRET2 based Gα Gβγ interaction assay

The plasmids encoding M2R was a gift from Dr. Roderick MacKinnon. The plasmids encoding Gα-RLuc8, Gβ1, and GFP2-Gγ1 were gifts from Dr. Bryan Roth. The plasmid encoding GFP2-Gγ2 was generated by replacing the Gγ1 sequence of pcDNA3.1-GGamma1-GFP2 by digestion with BamHI/XbaI and subsequent insertion of the Gγ2 sequence(MASNNTASIAQARKLVEQLKMEANIDRIKV-SKAAADLMAYCEAHAKEDPLLTPVPASENPFREKKFFCAIL). All plasmids were sequenced to ensure their identities.

The BRET2 assay was conducted as reported (Olsen et al., 2020). Cells were plated in 10 cm dishes at 2.5–3 million cells per dish the night before transfection. Cells were transfected using a 6:6:3:1 DNA ratio of receptor:Gα-RLuc8:Gβ:GFP2-Gγ (750:750:375:125 ng for 10 cm dishes). Transit 2020 was used to complex the DNA at a ratio of 3 μL Transit per μg DNA, in OptiMEM at a concentration of 10 ng DNA per μL OptiMEM. 16 h after transfection, cells were harvested from the plate using TrypLE and plated in poly-D-lysine-coated white, clear-bottom 96-well assay plates at a density of 30,000–35,000 cells per well.

8 h after plating in 96-well assay plates, media was replaced with 100 μL of cyclic peptide dilutions (Final cyclic peptide concentrations were indicated in Figures) in DMEM with 1% dialyzed FBS. 16 h after drug treatment at 37°C with 5% CO_2_, white backings were applied to the plate bottoms, and growth medium was carefully aspirated and replaced immediately with 60 μL of 1.67X drug dilutions in assay buffer (1× Hank’s balanced salt solution (HBSS) + 20 mM HEPES, pH 7.4), followed by a 10 μL addition of freshly prepared 50 μM coelenterazine 400a. After a 5 min equilibration period, cells were treated with 30 μL of 3.33X GPCR agonist or DMSO dilutions in assay buffer for an additional 5 min. Plates were then read in a TECAN Spark 20M plate reader with 395 nm (RLuc8-coelenterazine 400a) and 510 nm (GFP2) emission filters, at integration times of 1 s per well. Plates were read serially six times, and measurements from the fourth read were used in all analyses. BRET2 ratios were computed as the ratio of the GFP2 emission to RLuc8 emission.

#### Whole-cell voltage-clamp recordings

The plasmids encoding Gβ1-C Venus, Gγ2-N Venus, and GIRK4-NLuc were gifts from Dr. Roderick MacKinnon. Cells were plated in 6 well plate at 0.55 million cells per well the night before transfection. Cells were transfected β2AR (100 ng), Gβ1-C Venus (25 ng), Gγ2-N Venus (25 ng), GIRK4-NLuc (100 ng). 1.75 μL of Lipofectamine (2000) was used to complex the DNA in 88 μL of OptiMEM. Transfected cells were incubated at 37°C for 12 h. After 12 h, cells were plated on glass coverslips and incubated at 37°C for 12 h for electrophysiological recordings. Whole-cell voltage-clamp recordings were performed with an Axopatch 200B amplifier (Molecular Devices, San Jose, CA) in the whole-cell mode. The analog current signal was low-pass filtered at 5 kHz (Bessel) and digitized at 50 kHz with a Digidata 1550B digitizer (Molecular Devices, San Jose, CA). Digitized data was recorded using the software pClamp 10.7. Patch electrodes (resistance 2.0–4.0 MΩ) were pulled on a Sutter P-97 puller (Sutter Instrument Company, Novato, CA) from 1.5 mm outer diameter filamented borosilicate glass. Extracellular solution contained 140 mM NaCl, 5 mM KCl, 2 mM CaCl_2_,2 mM MgCl_2_, 10 mM D-glucose, 10 mM HEPES-NaOH (pH 7.4) (~330 mOsm). The extracellular solution was exchanged to high K^+^ solution containing 40 mM NaCl, 100 mM KCl, 2 mM CaCl_2_, 2 mM MgCl_2_, 10 mM D-glucose, 10 mM HEPES-NaOH (pH 7.4) (~330 mOsm). The pipette solution contained 13.5 mM NaCl, 140 mM K-aspartate, 1.6 mM MgCl_2_, 0.09 mM EGTA-K, 9 mM HEPES-KOH (pH 7.2) (~290 mOsm). 1% DMSO, 25 μM cpGD20 in 1% DMSO, or 25 μM cpGD20-F5A in 1% DMSO was added to the pipette solution before the experiments.

#### Chemical stability assay

These assays were conducted by Pharmaron Beijing CO., Ltd. Cyclic peptides working solutions were prepared at 10 μM in DMEM with 10% FBS (Avantor, Cat# 76,294–180) or human plasma (Pooled, Male & Female, BioIVT, Cat# HMN666664). The assays were performed in duplicate. Vials were incubated at 37 °C at 60 rpm in a water bath and taken at designated time points including 0, 480, 1080 and 1440 min. For each time point, the initiation of the reaction was staggered so all the time points were terminated with cold acetonitrile containing internal standards (IS, 100 nM alprazolam, 200 nM labetalol, 200 nM Imipramine and 2 μM ketoplofen) at the same time. Samples were vortexed then centrifuged at 4°C to remove proteins. The supernatants from centrifugation were diluted by ultra-pure H_2_O and used for LC-MS/MS analysis. All calculations were carried out using GraphPad Prism. Remaining percentages of parent compounds at each time point were estimated by determining the peak area ratios from extractedion chromatograms.

#### Chemical synthesis

Solid phase synthesis of cyclic peptides: Macrocyclic peptides (25 μmol scale) were synthesized by a standard Fmoc solid phase peptide synthesis method using a Syro Wave automated peptide synthesizer (Morimoto et al., 2012). After addition of a chloroacetyl group onto the N-terminal amide group (for the formation of cyclic peptide), peptides were cleaved from the NovaPEG Rink Amide resin (Novabiochem) by a solution of 92.5% trifluoroacetic acid (TFA), 2.5% 3,6-Dioxa-1,8-octanedithiol ethanedithiol (DODT), 2.5% triisopropylsilane (TIPS) and 2.5% water and precipitated by diethyl ether. To conduct the macrocyclization reaction, the peptide pellet was dissolved in 10 mL DMSO containing 10 mM tris(2-carboxyethyl)phosphine hydrochloride (TCEP), adjusted to pH > 8 by addition of triethylamine (TEA) and incubated at 25°C for 1 h. This cyclization reaction was quenched by acidification of the solution with TFA. The crude products were purified by reverse-phase HPLC (RP-HPLC) (Shimadzu) with a Chromolith RP-18 100–25 prep column. Molecular masses were verified by a time-of-flight mass spectrometer (Waters Xevo G2-XS), and the purity was verified by analytical HPLC on a Waters Acquity UPLC BEH C18 1.7 μm column.

General synthesis route of chloroalkane tagged cyclic peptides: In this work, we prepared a chloroalkane tag (ct) that has been previously used with the HaloTag system (Neklesa et al., 2011). Instead of using the Rink amide resin, peptides were synthesized using the Fmoc-Wang resin (Anaspec, AS-20058) to generate a carboxylate at the C-terminus. To cap the C-terminus with the chloroalkane tag (ct), 10 equiv of chloroalkane tag (ct), 5 equiv of HATU, and 20 equiv of DIPEA were dissolved in DMF and stirred for 1 h at room temperature. Crude peptides were purified by reverse-phase HPLC (Waters XBridge C18 column 5 μm particle size 30 × 250 mm, 5%–95% acetonitrile-water + 0.1% formic acid, 40 min, 20 mL/min) to afford the chloroalkane tagged peptides.

#### Characterization data for cyclic peptides

##### Mass spectrometry

GN13: HRMS (ESI): Calcd for (C_79_H_106_N_16_O_21_S + 2H)^2+^: 824.3798, Found: 824.3973.

GN13-E3A: HRMS (ESI): Calcd for (C_77_H_104_N_16_O_19_S + 2H)^2+^: 795.3770, Found: 795.3749.

GN13-I8A: HRMS (ESI): Calcd for (C_76_H_100_N_16_O_21_S + 2H)^2+^: 803.3563, Found: 803.3563.

GN13-W9A: HRMS (ESI): Calcd for (C_71_H_101_N_15_O_21_S + 2H)^2+^: 766.8587, Found: 766.8610.

cpGN13: HRMS (ESI): Calcd for (C_140_H_224_N_54_O_36_S + 3H)^3+^: 1090.9104, Found: 1090.9111.

GN13-E3Q-Biotin: HRMS (ESI): Calcd for (C_113_H_170_N_20_O_33_S_2_ + 2H)^2+^: 1200.5919, Found: 1200.5970.

ct-GN13-E3Q: HRMS (ESI): Calcd for (C_89_H_126_ClN_17_O_22_S + 2H)^2+^: 926.9415, Found: 926.9422.

GD20: HRMS (ESI): Calcd for (C_90_H_126_N_22_O_20_S + 2H)^2+^: 934.4698, Found: 934.4844.

cpGD20 (GD20-F10L): HRMS (ESI): Calcd for (C_87_H_128_N_22_O_20_S + 2H)^2+^: 917.4776, Found: 917.4901.

ct-GD20: HRMS (ESI): Calcd for (C_100_H_145_ClN_22_O_22_S + 2H)^2+^: 1037.5235, Found: 1037.5303.

ct-GD20-F10L: HRMS (ESI): Calcd for (C_97_H_147_ClN_22_O_22_S + 2H)^2+^: 1020.5313, Found: 1020.5193.

GD20-Biotin: HRMS (ESI): Calcd for (C_124_H_189_N_25_O_33_S_2_ + 2H)^2+^: 1311.1739, Found: 1311.1741.

cpGD20-Biotin: HRMS (ESI): Calcd for (C_121_H_191_N_25_O_33_S_2_ + 2H)^2+^: 1294.1817, Found: 1294.1805.

GD20-F5A: HRMS (ESI): Calcd for (C_84_H_122_N_22_O_20_S + 2H)^2+^: 896.4542, Found: 896.4604.

cpGD20-F5A: HRMS (ESI): Calcd for (C_81_H_124_N_22_O2_0_S + 2H)^2+^: 879.4620, Found: 879.4648.

GD20-R6A: HRMS (ESI): Calcd for (C_87_H_119_N_19_O_20_S + 2H)^2+^: 891.9378, Found: 891.9394.

GD20-W8A: HRMS (ESI): Calcd for (C_82_H_121_N_21_O_20_S + 2H)^2+^: 876.9487, Found: 876.9509

Absorbance was recorded at 280 nm (Figure S1D).

### QUANTIFICATION AND STATISTICAL ANALYSIS

All of the curves in Figures except those from the BLI experiments were fitted by GraphPad Prism. Raw kinetic data collected from the BLI experiments were processed with the Data Analysis software provided by the manufacturer. All the details can be found in the figure legends and in the Method details. The data collection and refinement statistics of the crystal structures can be found in Tables S1 and S2 (related to Figures 3 and 5, see also Figures S3 and S5).

## Supplementary Material

MMC1

MMC2

3

## Figures and Tables

**Table:** KEY RESOURCES TABLE

REAGENT or RESOURCE	SOURCE	IDENTIFIER
Bacterial and virus strains		

*Escherichia coli* BL21(DE3)	Invitrogen	Cat# C600003
MAX Efficiency DH10Bac Competent Cells	Thermo Fisher Scientific	Cat# 10,361,012

Chemicals, peptides, and recombinant proteins		

GDP	Sigma-Aldrich	Cat# G7127–100MG
GTP	Sigma-Aldrich	Cat# 11,140,957,001
ATP	Discoverx	Cat# 90–0099
Guanosine 5’-[β,γ-imido]triphosphate (GNP, GppNHp)	Axorra	Cat# JBS-NU-401–50
100X GTPγS, 10mM	EMD Millipore	Cat# 20–176
Guanosine 5ʹ-Diphosphate, Trisodium Salt, [8,50–3H]-, Specific Activity: 25–50Ci (0.925–1.85TBq)/mMole, 250 μCi (9.25MBq)	Perkin-Elmer	Cat# NET966250UC
GTP, [γ−32P]-6000 Ci/mmol 10 mCi/ml Lead, 250 μCi	Perkin-Elmer	Cat# NEG004Z250UC
GTPγS, [35S]-1250 Ci/mmol, 12.5 mCi/ml, 250 μCi	Perkin-Elmer	Cat# NEG030H250UC
Forskolin	Cayman Chemical Company	Cat# 11,018: 50 mg
Isoproterenol Hydrochloride	TCI	Cat# I0260
Acetylcholine Chloride	Selleckchem	Cat# S1805
Activated Charcoal Norit	Sigma-Aldrich	Cat# 53,663–250G
Cytoscint-ES liquid scintillation cocktail	MP Biomedicals	Cat# 0,188,245,301
Acrylonitrile	Sigma-Aldrich	Cat# 110,213–5ML
cOmplete Protease Inhibitor Cocktail	Sigma-Aldrich	Cat# 5,056,489,001
Carbenicillin	Goldbio	Cat# C-103–100
Kanamycin	Goldbio	Cat# K-120–25
IPTG	Goldbio	Cat# I2481C100
DTT	Goldbio	Cat# DTT10
Biotin	Sigma-Aldrich	Cat# B4501–5G
M-MLV reverse transcriptase	Promega	Cat# 3683
acetylated BSA	Nacalai Tesque	Cat# 01,278–44
TrypLE™ Express Enzyme (1X), no phenol red	Fisher Scientific	Cat# 12,604,013
PBS, pH 7.4	Thermo Fisher Scientific	Cat# 10,010,049
3-Isobutyl-1-methylxanthine, BioUltra, ≥99% (IBMX)	Sigma-Aldrich	Cat# I7018
DMSO sterile filtered	Sigma-Aldrich	Cat# D2650
ct-TAMRA	Promega	Cat# G8251
Coelenterazine-400a (Nanolight Technology)	Prolume Ltd	Cat# 340–1
BSA, Fraction V, low Heavy Metals	EMD Millipore	Cat# 12,659–100GM

Critical commercial assays		

LANCE Ultra cAMP Detection Kit	Perkin-Elmer	Cat# TRF0263
GTPase Colorimetric Assay Kit 480 Tests	Innova Biosciences	Cat# 602–0121
Pierce™ BCA® Protein Assay Kits and Reagents, Thermo Scientific, BCA	Fisher Scientific	Cat# PI23227
Streptavidin XL665	Cisbio	Cat# 610SAXLF
Anti-6His-Tb cryptate	Cisbio	Cat# 61HI2TLF
Green Up cADDis cAMP Assay Kit	Montana Molecular	Cat# U0200G

Deposited data		

GppNHp-bound Gαs in complex with the cyclic peptide inhibitor GN13	This paper	PDB: 7BPH
GDP-bound Gαs in complex with the cyclic peptide inhibitor GD20	This paper	PDB: 7E5E
GTPγS-bound Gαs	Sunahara et al. (1997)	PDB: 1AZT
GTPγS-bound Gαs in complex with adenylyl cyclase	Tesmer et al. (1997)	PDB: 1AZS
GDP-bound Gαs in complex with Gβγ	Liu et al. (2019)	PDB: 6EG8
GDP•AlF4^-^-bound Gα13 in complex with PRG rgRGS domain	Chen et al. (2008)	PDB: 3CX7
GDP•AlF4^-^-bound Gαq in complex with RGS8	Taylor et al. (2016)	PDB: 5DO9
GDP•AlF4^-^-bound Gαi in complex with KB1573	Johnston et al. (2006)	PDB: 2G83
GDP-bound Gα13 in complex with PRG rgRGS domain	Chen et al. (2008)	PDB: 3CX6
GDP-bound Gαq in complex with Gβγ and YM-254890	Nishimura et al. (2010)	PDB: 3AH8
GDP-bound Gαi in complex with Gβγ	Wall et al. (1995)	PDB: 1GP2
GDP•AlF4^-^-bound Gα(t/i) in complex with RGS9 and PDEγ	Slep et al. (2001)	PDB: 1FQJ
Gαs/Gβγ/β2AR/Nb35 complex	Rasmussen et al. (2011)	PDB: 3SN6

Experimental models: Cell lines		

Sf9 cells	Thermo Fisher Scientific	Cat# 12,659,017
Halo-Tag-GFP-Mito expressing HeLa cells	J. Kritzer (Tufts University) (Peraro et al., 2018)	N/A
HEK293 cells	ATCC	Cat# CRL-1573
(Parent) HEK293 cells	A. Inoue (Tohoku University) (Stallaert et al., 2017)	N/A
GNAS KO HEK293 cells (CL4)	A. Inoue (Tohoku University) (Stallaert et al., 2017)	N/A

Recombinant DNA		

Gαs(WT) cloned into a modified pET15b vector	(Hu and Shokat, 2018)	N/A
Gαs(Q227L) cloned into a modified pET15b vector	This study	N/A
Human ADCY2 (residues 871–1082) cloned into a modified pET15b vector	(Hu and Shokat, 2018)	N/A
Mouse ADCY5(D628E/S645R) (residues 443–659) cloned into a pET29b vector	(Hu and Shokat, 2018)	N/A
HumanGNB1(WT) and GNG2(C68S) cloned into a modified pFastBac Dual vector	(Hu and Shokat, 2018)	N/A
Avi-Gαs(WT) cloned into a modified pET15b vector	This study	N/A
Avi-Gαi1(WT) cloned into a modified pET15b vector	This study	N/A
Avi-Gαs(S275L) cloned into a modified pET15b vector	This study	N/A
pcDNA3 Gαs(WT)-HA	This study	N/A
pcDNA3 Gαs(S275L)-HA	This study	N/A
pcDNA3 Gαsi1(WT)-EE-tagged	G. Peng (UCSF)	N/A
SSF-β2AR	B. Barsi-Rhyne (UCSF)	N/A
pCEH-Sero-SNAP-hM2R	R. Mackinnon (The Rockefeller University)	N/A
Gβ1-C Venus	R. Mackinnon (The Rockefeller University)	N/A
Gγ2-N Venus	R. Mackinnon (The Rockefeller University)	N/A
GIRK4-NLuc	R. Mackinnon (The Rockefeller University)	N/A
pcDNA3.1-Beta1	Olsen et al. (2020)	Addgene plasmid # 140,987
pcDNA3.1-GGamma1-GFP2	Olsen et al. (2020)	Addgene plasmid # 140,989
pcDNA3.1-GGamma2-GFP2	This paper	N/A
pcDNA5/FRT/TO-GAlphai1-RLuc8	Olsen et al. (2020)	Addgene plasmid # 140,973
pcDNA5/FRT/TO-GAlphasS-RLuc8	Olsen et al. (2020)	Addgene plasmid # 140,980

Software and algorithms		

GraphPad Prism	GraphPad Software	https://www.graphpad.com/scientific-software/prism/
CCP4	Winn et al. (2011)	http://www.ccp4.ac.uk/
Phenix	Adams et al. (2010)	https://www.phenix-online.org/
Coot	Emsley et al. (2010)	https://www2.mrc-lmb.cam.ac.uk/%20personal/pemsley/coot/
Excel	Microsoft	https://www.microsoft.com/en-us/
Word	Microsoft	https://www.microsoft.com/en-us/
Illustrator	Adobe	https://www.adobe.com/products/illustrator.html
Pymol	The PyMOL Molecular Graphics System, Version 1.8 Schrödinger, LLC.	https://pymol.org/2/
UCSF Chimera	Pettersen et al. (2004)	https://www.cgl.ucsf.edu/chimera

Other		

TALON Metal Affinity Resin	Clontech Laboratories	Cat# 635,503
SOURCE 15Q, 200 mL	GE Healthcare	Cat# 17–0947-05
Superdex 200 Increase 10/300 GL	GE Healthcare	Cat# 28–9909-44
Sephadex G-25	GE Healthcare	Cat# 17,003,201
Dynabeads M280 streptavidin magnetic beads	Thermo Fisher Scientific	Cat# 11206D
Transit 2020	Fisher Scientific	Cat# MIR5404
Lipofectamine 2000 Transfection Reagent	Thermo Fisher Scientific	Cat# 11,668,019
Opti-MEM™ I Reduced Serum Medium	Fisher Scientific	Cat# 31–985-062
Mixed cellulose membrane	EMD Millipore	Cat# GSWP02500
Streptavidin biosensors	Molecular Devices	Cat# 18–5019
Sf-900 III SFM	Thermo Fisher Scientific	Cat# 12,658,027
OptiPlate-384, White Opaque 384-well Microplate	PerkinElmer	Cat# 6,007,290
Greiner 384well, black, flat bottom polypropylene plates	Millipore Sigma	Cat# M1937–32EA
96-well Flat Clear Bottom Black Polystyrene Microplates	Corning	Cat# 3340
poly-D-lysine-coated white, clear-bottom 96-well assay plates	Greiner Bio-One	Cat# 655,944
White Adhesive Bottom Seal	Perkin Elmer	Cat# 6,005,199
Dounce tissue grinder set	Millipore Sigma	Cat# D8938–1SET
Spark 20 M plate reader	TECAN	N/A
Synergy H4 Hybrid Microplate Reader	BioTek	N/A
Octet RED384	ForteBio	N/A
LS 6500 Multi-Purpose Scintillation Counter	Beckman Coulter	N/A
Axopatch 200B amplifier	Molecular Devices	N/A
Digidata 1550B digitizer	Molecular Devices	N/A
Sutter P-97 puller	Sutter Instrument Company	N/A
Syro Wave automated peptide synthesizer	Biotage	N/A

## Highlights

- Discovery of nucleotide-state-selective cyclic peptide binders for Gαs
- Co-crystal structures reveal G protein class specificity of the cyclic peptides
- Inhibition of the Gαs ON-state reduces isoproterenol-stimulated cAMP production
- Sequestering the Gαs OFF-state prolongs Gβγ activation upon receptor activation
