# Supplementary material for: State-selective modulation of heterotrimeric Gαs signaling with macrocyclic peptides
Source: Cell. Author manuscript; Available in PMC 2022 Dec 13. (PMC9747239; doi:10.1016/j.cell.2022.09.019)
Supplement: MMC1 [file NIHMS1837800-supplement-MMC1.pdf]

Cell, Volume *185*

Supplemental information

State-selective modulation of heterotrimeric Gαs signaling with macro-cyclic peptides

Shizhong A. Dai, Qi Hu, Rong Gao, Emily E. Blythe, Kouki K. Touhara, Hayden Peacock, Ziyang Zhang, Mark von Zastrow, Hiroaki Suga, and Kevan M. Shokat

Table S1: Data collection and refinement statistics for the Gαs/GppNHp/GN13 complex. Related to Figure 3

	Gαs/GppNHp/GN13 complex
**Data collection**	
Space group	P 21 21 21
Cell dimensions	
a, *b*, *c* (Å)	68.905, 78.332, 80.043
α, *β*, *γ* (°)	90, 90, 90
Resolution (Å)	50.00-1.57 (1.60-1.57)[a]
R_{merge}, R_{meas}, and R_{pim}	0.074 (1.040), 0.086 (0.972), 0.023 (0.405)
$I/\sigma(I)$	27.3 (1.32)
$CC_{1/2}$	0.997 (0.783)
Completeness (%)	96.9 (71.2)
Total reflections	58703
Unique reflections	57627
Redundancy	12.4 (4.0)
**Refinement**	
Resolution (Å)	43.45-1.574
No. reflections	51161
R_{work}	0.1970
R_{free}	0.2291
No. atoms	
Protein	3079
Ligand/ion (specify/describe)	44
Water	132
B factors	
Protein	21.98
Ligand/ion	13.41
Water	22.43
R.m.s. deviations	
Bond lengths (Å)	0.014
Bond angles (°)	1.39
Ramachandran analysis	
Favored (%)	98.91
Allowed (%)	0.82
Outliers (%)	0.27
Rotamer outliers (%)	0.60
Clashscore	3.57

[a] Values in parentheses are for highest-resolution shell.

Table S2: Data collection and refinement statistics for the Gαs/GDP/GD20 complex. Related to Figure 5

	Gαs/GDP/GD20 complex
**Data collection**	
Space group	P1
Cell dimensions	
a, *b*, *c* (Å)	58.105, 81.771, 76.912
α, *β*, *γ* (°)	81.266, 83.844, 90.698
Resolution (Å)	50.00-1.95 (1.98-1.95)[a]
R_{merge}, R_{meas}, and R_{pim}	0.081 (1.202), 0.113 (1.260), 0.059 (0.762)
$I/\sigma(I)$	11.40 (0.815)
$CC_{1/2}$	0.991 (0.422)
Completeness (%)	97.0 (87.7)
Total reflections	99153
Unique reflections	95974
Redundancy	3.4 (2.0)
**Refinement**	
Resolution (Å)	48.54-1.95
No. reflections	82224
R_{work}	0.2178
R_{free}	0.2580
No. atoms	
Protein	11279
Ligand/ion	132
Water	329
B factors	
Protein	29.36
Ligand/ion	18.22
Water	21.27
R.m.s. deviations	
Bond lengths (Å)	0.004
Bond angles (°)	0.70
Ramachandran analysis	
Favored (%)	97.45
Allowed (%)	2.25
Outliers (%)	0.30
Rotamer outliers (%)	0.98
Clashscore	3.86

[a] Values in parentheses are for highest-resolution shell.

Table S3: Kinetics analysis of cyclic peptides-Gαs interaction by BLI. Related to Figure 2D, 4C, S2, S5 and S7

	K_D (nM)	K_{on} ($M^{-1}s^{-1}$)	K_{off} (s^{-1})
GN13/GppNHp/Gαs	190 ± 16	$2.71 \times 10^5 \pm 2.13 \times 10^4$	$5.13 \times 10^{-2} \pm 1.31 \times 10^{-3}$
GD20/GDP/Gαs	31.4 ± 0.7	$1.09 \times 10^5 \pm 1.28 \times 10^3$	$3.43 \times 10^{-3} \pm 6.97 \times 10^{-5}$
cpGD20/GDP/Gαs	14.5 ± 0.4	$2.47 \times 10^5 \pm 3.71 \times 10^3$	$3.58 \times 10^{-3} \pm 8.50 \times 10^{-5}$

Table S4: Chemical stability of Gαs binding cyclic peptides in DMEM with 10% FBS. Related to STAR Methods

	Half-life (hour) [a]
GN13	>76
GD20	>142
cpGD20	37.37 - 50.32

[a] Values represent 95% confidence intervals
The data were analyzed from two independent replicates.

Table S5: Plasma stability of Gαs binding cyclic peptides. Related to STAR Methods

	Half-life (hour) [a]
GN13	>82
GD20	16.37 - 27.13
cpGD20	7.16 - 12.74

[a] Values represent 95% confidence intervals
The data were analyzed from two independent replicates.
