# Supplementary material for: State-selective modulation of heterotrimeric Gαs signaling with macrocyclic peptides
Source: Cell. Author manuscript; Available in PMC 2022 Dec 13. (PMC9747239; doi:10.1016/j.cell.2022.09.019)
Supplement: 3 [file NIHMS1837800-supplement-3.pdf]

Supplemental figures

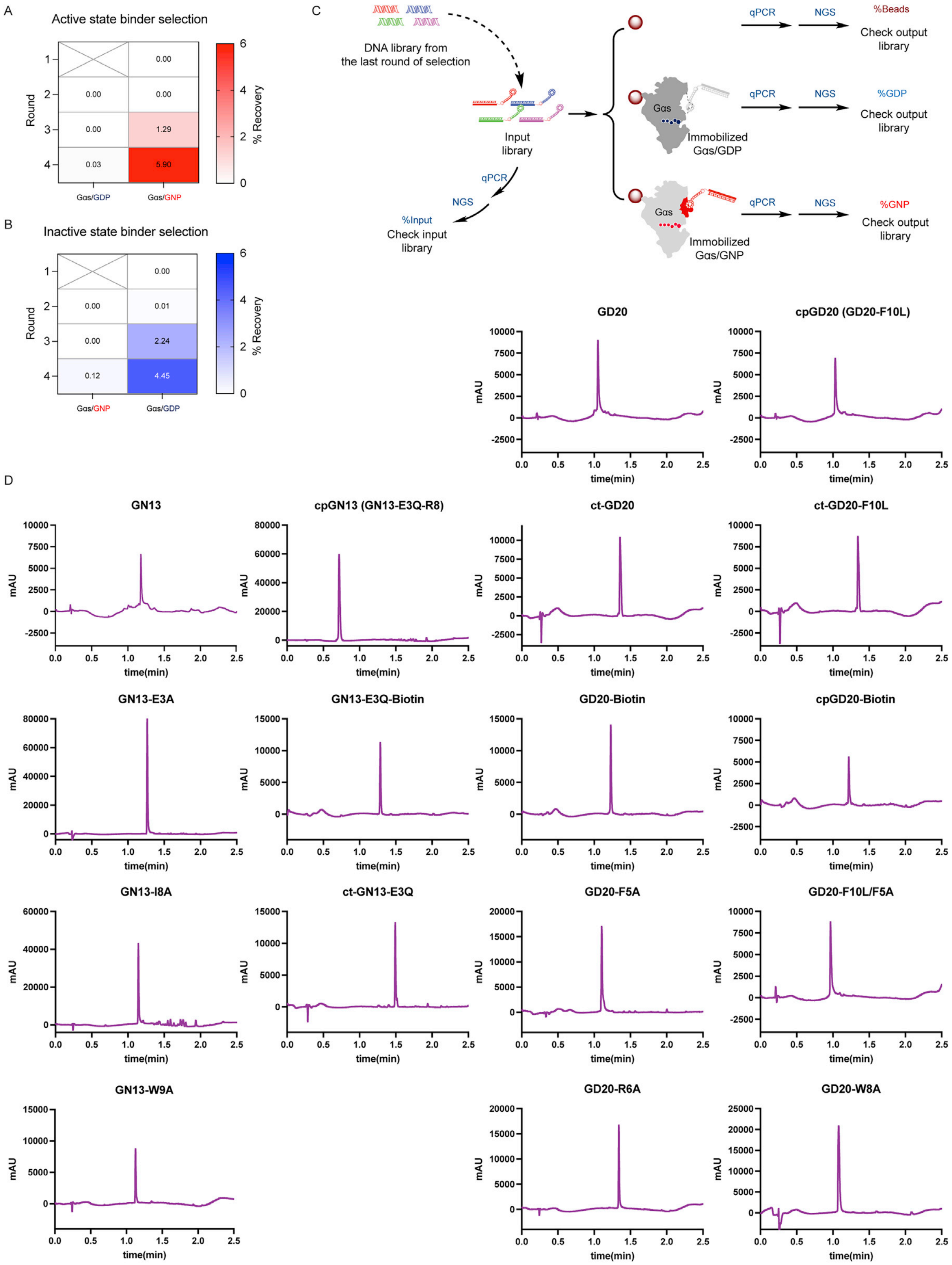

(legend on next page)

Figure S1. RaPID selection of state-selective Gαs binding cyclic peptides, related to Figure 1

(A and B) The percentage of enriched peptide-mRNA-cDNA complex in the input library after each selection was quantified by qPCR. Cyclic peptides that bind to GNP-bound (A) or GDP-bound (B) Gαs were enriched through R1-R4. To ensure a maximum library diversity at the initial stage of selection, negative selection was not included in the first round of selection.

(C) Comparison selection. DNA sequences of cyclic peptide binders from the R4 pools were quantified and identified by qPCR and NGS. A peptide-mRNA-cDNA complex library was produced based on the above-mentioned DNA sequences and equally split into three fractions. Binding of each individual peptide-mRNA-cDNA complex to blank, GDP-bound Gαs-immobilized or GNP-bound Gαs-immobilized beads was quantified by qPCR and NGS, respectively.

(D) Analytical HPLC Traces of resynthesized cyclic peptides. Absorbance was recorded at 280 nm.

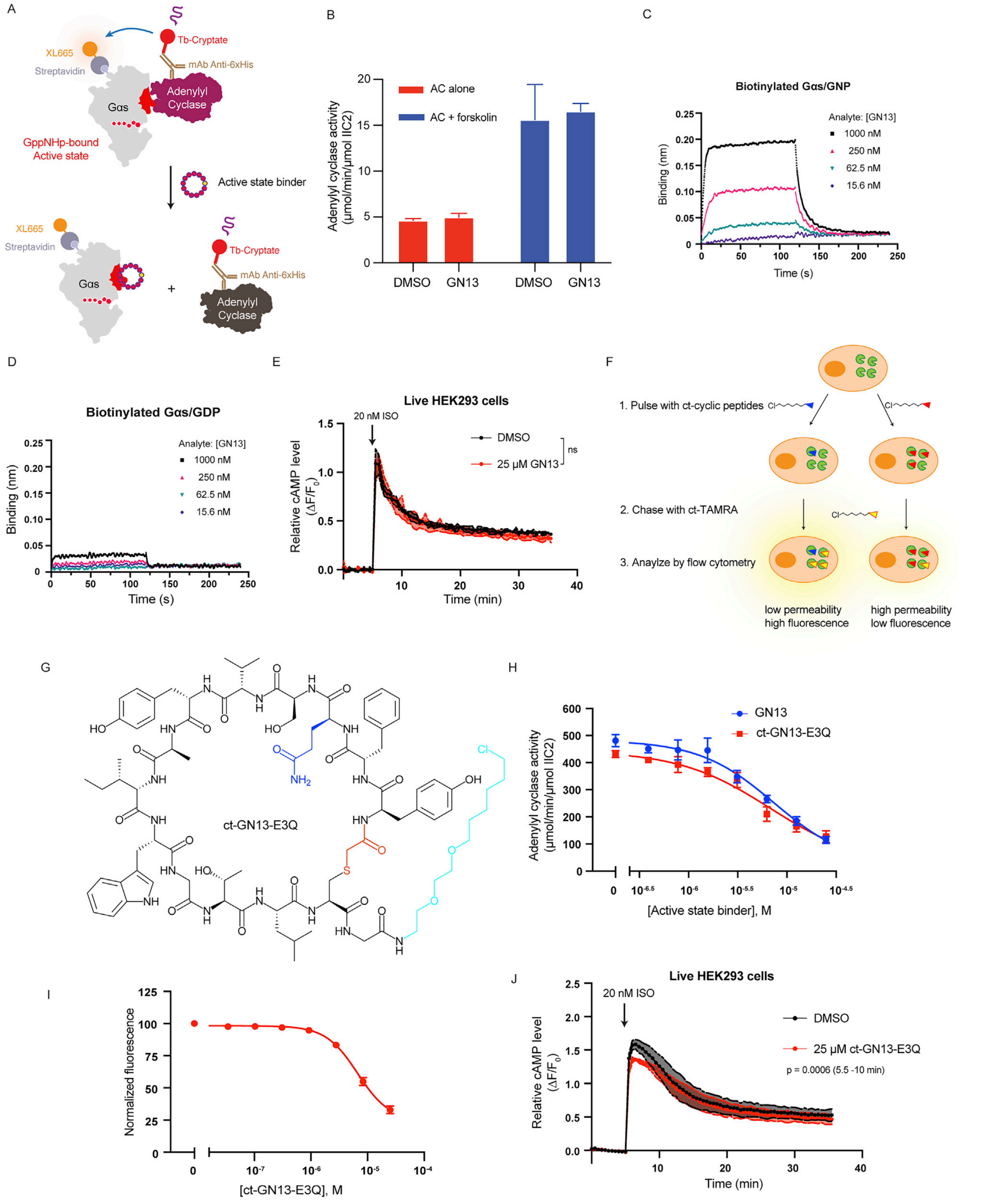

(legend on next page)

Figure S2. Gαs active-state inhibitor GN13 inhibits Gαs-mediated adenylyl cyclase activation, related to Figure 2

(A) Illustration of active-state binders inhibiting PPI between Gαs/GNP and AC.(B) GN13 did not directly inhibit the intrinsic or forskolin-mediated AC activity in the absence of Gαs. Mean ± SE, n = 3.

(C and D) Binding kinetics of GN13 to GNP-bound (C) or GDP-bound (D) Gαs were quantified using bio-layer Interferometry (BLI). Biotinylated Gαs proteins were immobilized to give a relative intensity of 2–3 nm on streptavidin biosensors. Association (t = 0-120 s) and dissociation (t = 120-240 s) cycles of compounds were started by dipping sensors into cyclic peptide solutions and control buffer. Binding signals were reference-subtracted. The assay was performed in duplicate, and the data represent one of the two replicates.

(E) Pretreament with GN13 for 24 h did not inhibited ISO-stimulated cAMP production in live HEK293 cells. Mean ± SD, n = 3. Two-tailed unpaired t tests (data after 5min). ns $p > 0.05$.

(F) Illustration of the chloroalkane penetration assay (CAPA).

(G) Structure of ct-GN13-E3Q. E3Q is colored blue. The ct tag is colored cyan.

(H) Activation of AC by Gαs was inhibited by both GN13 and ct-GN13-E3Q in a dose-dependent manner. Mean ± SE, n = 3.

(I) CAPA cell permeability assay result of ct-GN13-E3Q. Mean ± SD, n = 3.

(J) Pretreament with ct-GN13-E3Q for 24 h slightly inhibited ISO-stimulated cAMP production in live HEK293 cells. Mean ± SD, n = 3. Two-tailed unpaired t tests (data between 5.5 and 10 min), $p < 0.05$ was considered significant.

CellPress
OPEN ACCESS

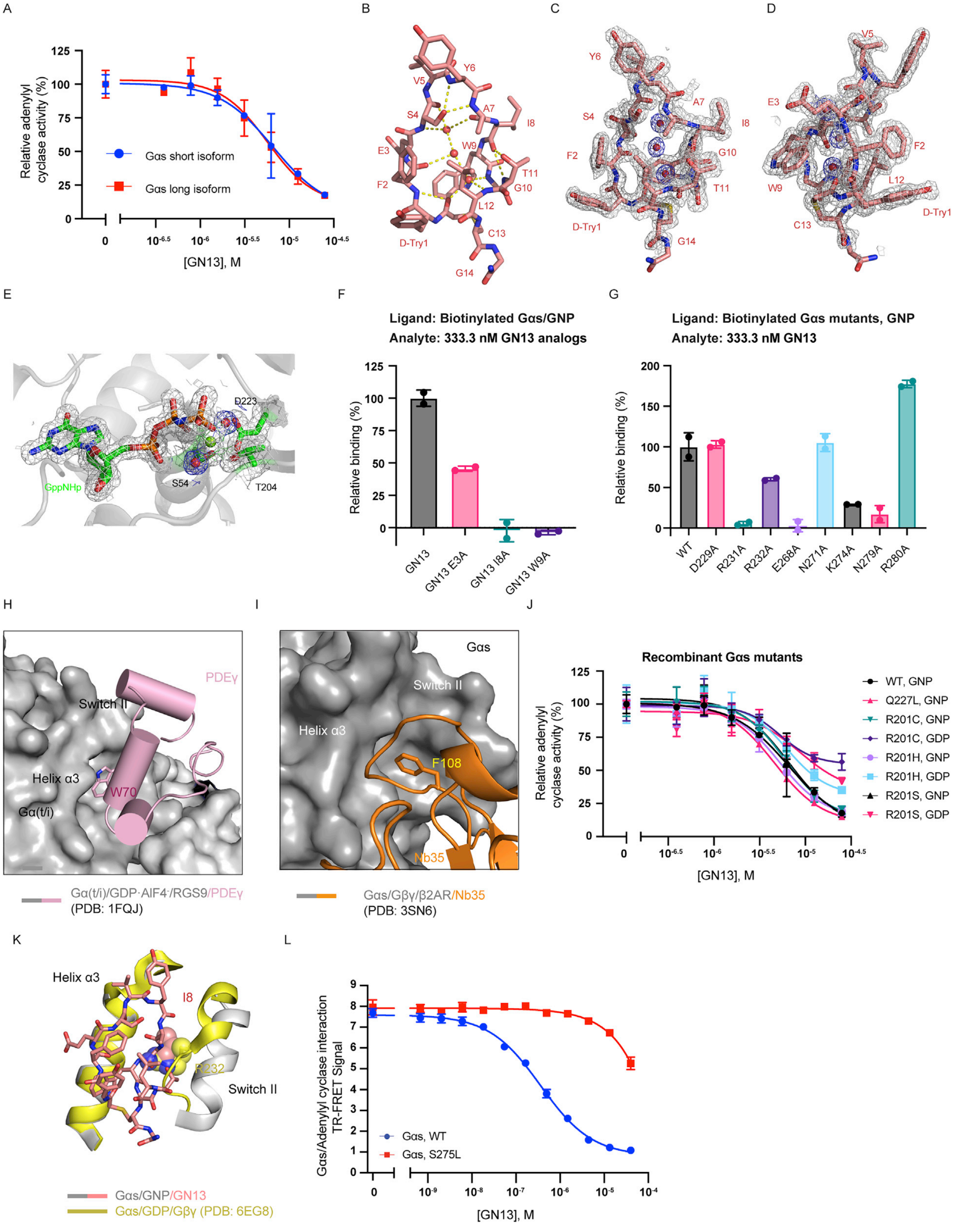

(legend on next page)

Figure S3. GN13 specifically inhibits Gαs through binding to a crystallographically defined pocket, related to Figure 3

(A) Activation of AC by both short and long isoforms of Gαs was inhibited by GN13. AC activity was normalized to DMSO control group (100%). Mean ± SD, n = 3.
(B) GN13 adopts a highly ordered three-dimensional structure through an H-bond network. GN13 is shown as salmon sticks. Three water molecules with well-defined electron density are shown as red spheres. H-bonds are represented by yellow dash lines.
(C and D) Electron density map of GN13. The *2mFo-DFc* electron density map of the structure is contoured at 1.0 σ and colored gray (GN13) and blue (Water), respectively.
(E) Electron density map of GNP. GNP and the side chains of S54, T204 and D223 are shown as sticks. The Mg^{2+} and two water molecules coordinated with the Mg^{2+} are shown as green and red spheres, respectively. The *2mFo-DFc* electron density map of the structure is contoured at 1.0 σ.
(F) Binding of GN13 analogs to WT Gαs/GNP were quantified using BLI. Biotinylated WT Gαs/GNP was immobilized to give a relative intensity of 2–3 nm on streptavidin biosensors, following the same association/dissociation cycles descried in Figure S2C. Binding signals were double referenced and normalized to Gαs loading and GN13/Gαs binding signal. Mean ± SD, n = 2.
(G) Binding of GN13 to different GNP-bound Gαs mutants were quantified using BLI. Biotinylated GNP-bound Gαs proteins were immobilized to give a relative intensity of 2–3 nm on streptavidin biosensors, following the same association/dissociation cycles descried in Figure S2C. Binding signals were double referenced and normalized to Gαs loading and GN13/WT Gαs binding signal. Mean ± SD, n = 2.
(H) Structure of the GDP•$AlF4^-$-bound Gα(t/i)/RGS9/PDEγ complex (PDB: 1FQJ). A critical tryptophan residue from PDEγ (pink, cartoon) engages the hydrophobic pocket between the switch II region and the α3 helix. Gα(t/i) and RGS9 are shown as surface. PDEγ is shown as cartoon.
(I) Structure of the Gαs/Gβγ/β2AR/Nb35 complex (PDB: 3SN6). A critical phenylalanine residue from Nb35 (orange, cartoon) engages the hydrophobic pocket between the switch II region and the α3 helix. Gαs is shown as surface. Nb35 is shown as cartoon.
(J) Activation of AC by Gαs oncogenic mutants was inhibited by GN13. AC activity was normalized to DMSO control group (100%). Mean ± SD, n = 3.
(K) Structural basis for nucleotide-state-selective binding of GN13 to Gαs. In Gαs/GDP (yellow), switch II is partially disordered, which disrupts polar contacts with GN13 and creates extensive steric hindrance. In particular, R232 of switch II (shown in space filling) is predicted to create a steric clash with I8 of GN13.
(L) GN13 inhibited PPI between Gαs WT and AC. This inhibitory effect was significantly diminished by the S275L mutation (red). Mean ± SD, n = 3.

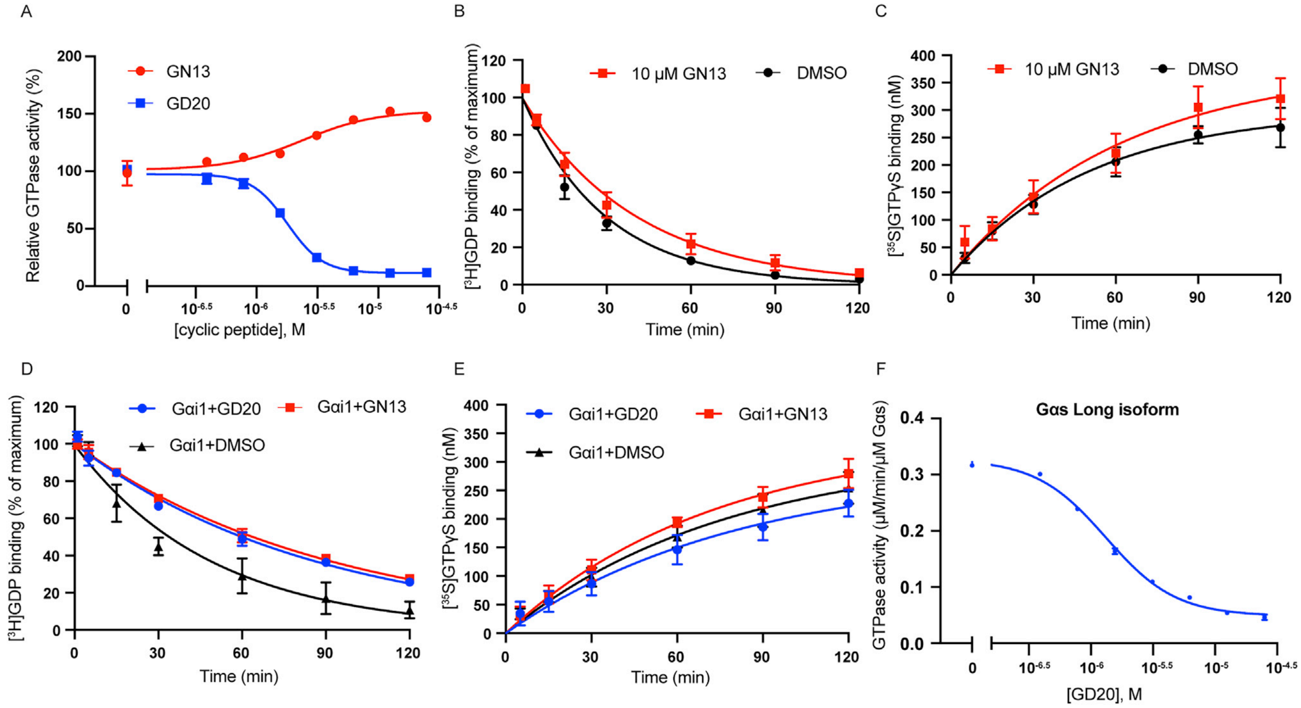

Figure S4. GN13 and GD20 modulate Gαs GTPase activity in a Gαs-specific manner, related to Figure 4

(A) Gαs steady-state GTPase activity was modulated by GN13 and GD20. Mean ± SD, n = 2.

(B) GDP dissociation from Gαs in the presence (red) or absence (black) of 10 μM GN13 were measured. Mean ± SD, n = 3.

(C) GTPγS binding to Gαs in the presence (red) or absence (black) of 10 μM GN13 were measured. Mean ± SD, n = 3.

(D) GDP dissociation from Gαi1 in the presence of 10 μM GN13 (red), or 10 μM GD20 (blue) or DMSO (black) were measured. Mean ± SD, n = 3.

(E) GTPγS binding to Gαi1 in the presence of 10 μM GN13 (red), or 10 μM GD20 (blue) or DMSO (black) were measured. Mean ± SD, n = 3.

(F) Steady-state GTPase activities of short and long isoforms of Gαs were inhibited by GD20. Mean ± SD, n = 2.

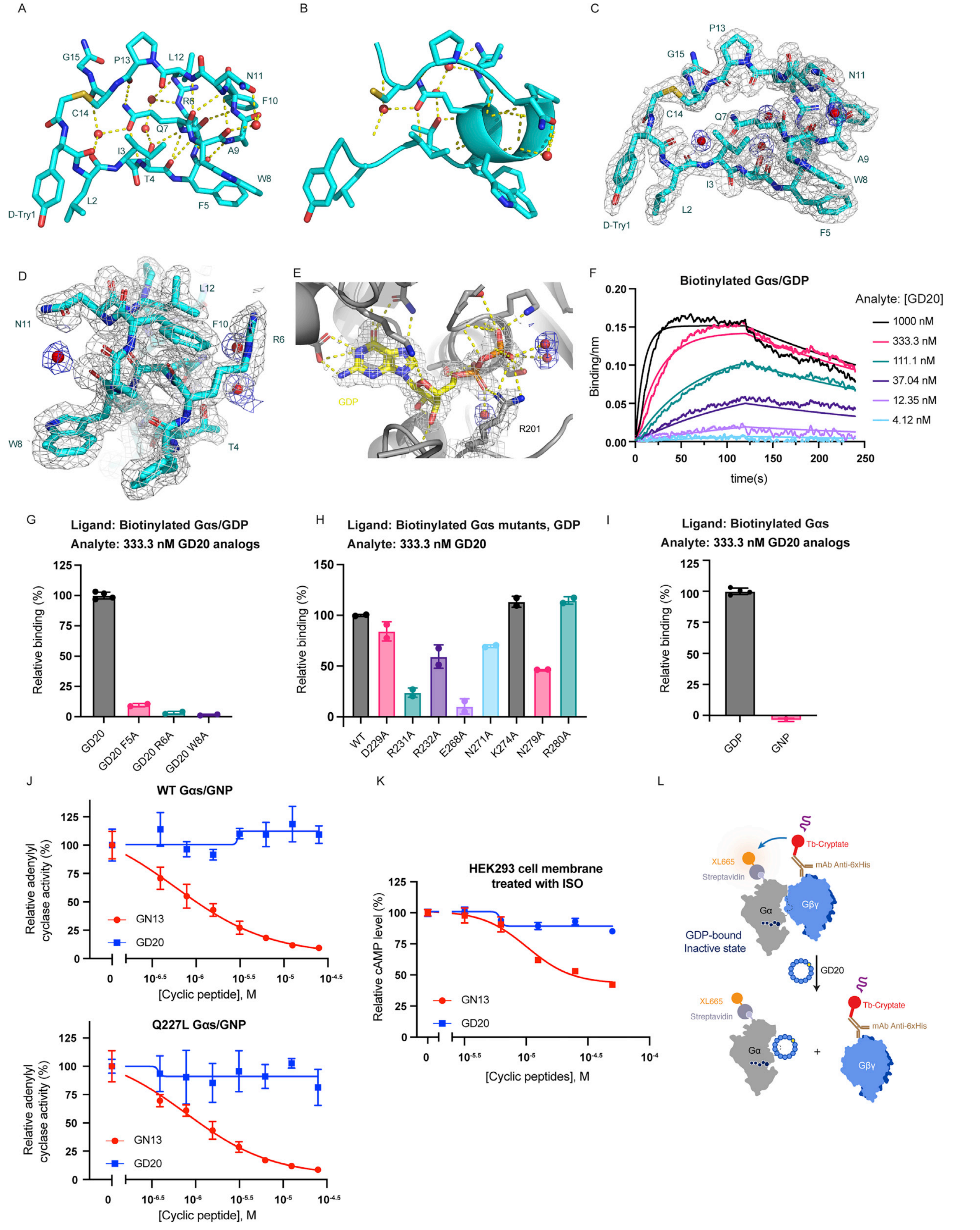

(legend on next page)

Figure S5. GD20 specifically inhibits Gαs through binding to a crystallographically defined pocket, related to Figure 5

(A and B) GD20 adopts a highly ordered three-dimensional structure through an H-bond network. GD20 is shown as cyan sticks (A) or cartoon (B). Four water molecules with well-defined electron density are shown as red spheres. H-bonds are represented by yellow dash lines.

(C and D) Electron density map of GD20. The *2mFo-DFc* electron density map of the structure is contoured at 1.0 σ and colored gray (GD20) and blue (Water), respectively.

(E) Electron density map of GDP. GDP and the side chain of R201 are shown as sticks. The Mg^{2+} and two water molecules coordinated with the Mg^{2+} are shown as green and red spheres, respectively. The *2mFo-DFc* electron density map of the structure is contoured at 1.0 σ.

(F) Binding kinetics of GD20 to WT Gαs/GDP were quantified using BLI. Biotinylated WT Gαs/GDP was immobilized to give a relative intensity of 2–3 nm on streptavidin biosensors, following the same association/dissociation cycles descried in Figure S2C. Binding signals were reference-subtracted. The assay was performed in duplicate, and the data represent one of the two replicates.

(G) Binding of GD20 analogs to WT Gαs/GDP were quantified using BLI. Biotinylated WT GDP-bound Gαs proteins were immobilized to give a relative intensity of 2–3 nm on streptavidin biosensors, following the same association/dissociation cycles descried in Figure S2C. Binding signals were double referenced and normalized to Gαs loading and GD20/Gαs binding signal. Mean ± SD, n = 2.

(H) Binding of GD20 to different GDP-bound Gαs mutants were quantified using BLI. Biotinylated GDP-bound Gαs proteins were immobilized to give a relative intensity of 2–3 nm on streptavidin biosensors, following the same association/dissociation cycles descried in Figure S2C. Binding signals were double referenced and normalized to Gαs loading and GDP/WT Gαs binding signal. Mean ± SD, n = 2.

(I) Binding of GD20 analogs to WT GDP-bound or GNP-bound Gαs were quantified using BLI. Biotinylated WT Gαs proteins were immobilized to give a relative intensity of 2–3 nm on streptavidin biosensors, following the same association/dissociation cycles descried in Figure S2C. Binding signals were double referenced and normalized to Gαs loading and GD20/GDP-bound Gαs binding signal. Mean ± SD, n = 2.

(J) Activation of AC by GNP-bound WT Gαs or GNP-bound Gαs oncogenic mutant Q227L were inhibited by GN13 but not GD20. AC activity was normalized to DMSO control group (100%). Mean ± SD, n = 3.

(K) GD20 did not inhibit ISO-stimulated Gαs activation in HEK293 cell membranes. Mean ± SD, n = 3.

(L) Illustration of inactive-state binders inhibiting PPI between Gαs/GDP and Gβγ(C68S).

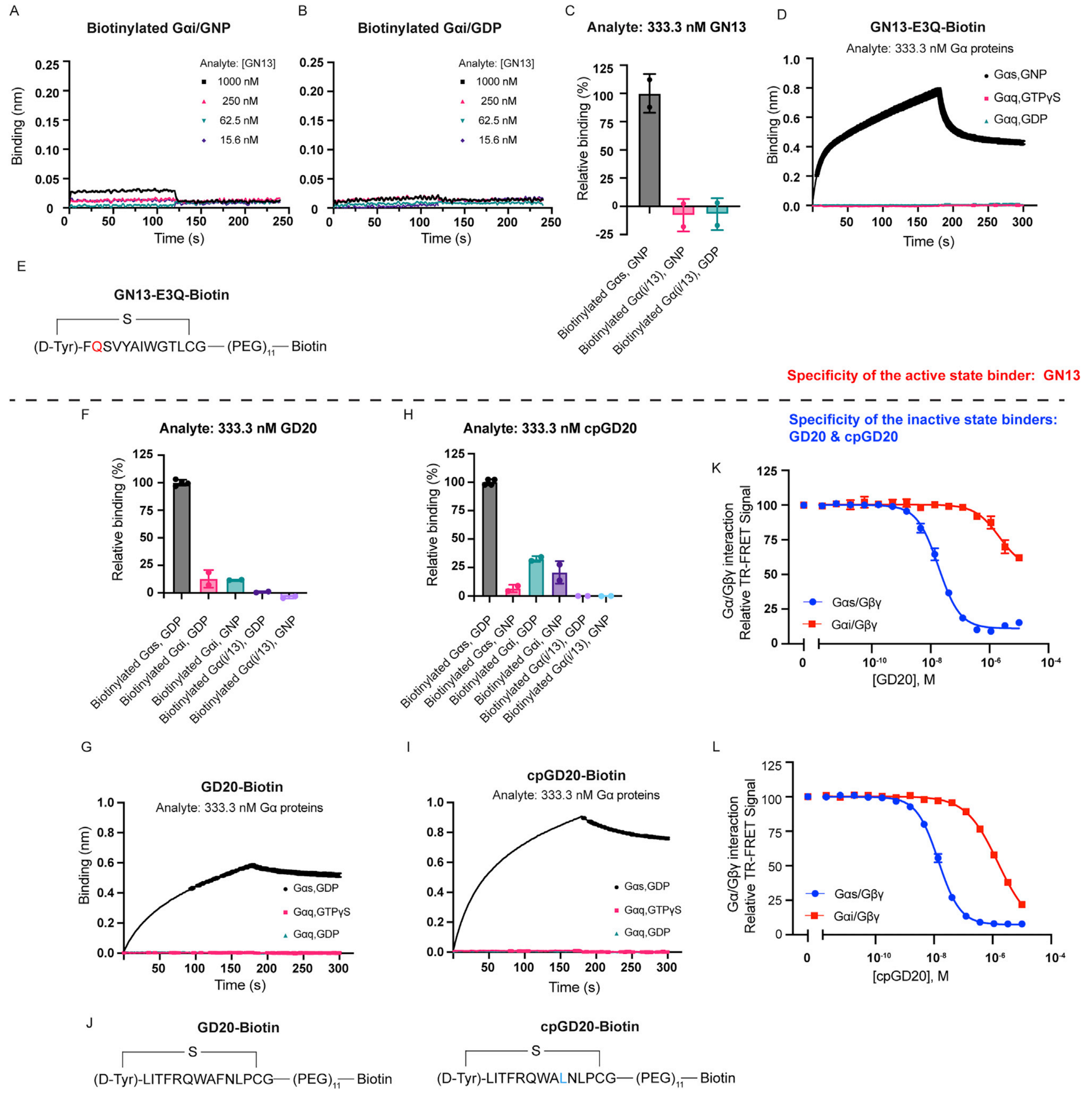

Figure S6. G protein class-specificity of GN13 and GD20, related to Figure 6

(A and B) Binding kinetics of GN13 to Gαi were quantified using BLI. Biotinylated Gαi proteins were immobilized to give a relative intensity of 2–3 nm on streptavidin biosensors, following the same association/dissociation cycles descried in Figure S2C. Binding signals were reference-subtracted. The assay was performed in duplicate, and the data represent one of the two replicates.

(C) Binding of GN13 to Gαs and Gα(i/13) were quantified using BLI. The substitution of the N-terminal helix of Gαi1 for the corresponding region of Gα13 generated soluble chimeric Gα(i/13) protein. The N-terminal helix substitution is far away from GN13 binding interface, therefore will not influence its binding. Biotinylated Gα proteins were immobilized to give a relative intensity of 2–3 nm on streptavidin biosensors, following the same association/dissociation cycles descried in Figure S2C. Binding signals were double referenced and normalized to Gα protein loading and GN13/GNP-bound Gαs binding signal. Mean ± SD, n = 2.

(D) The Avi tagged Gαq was insoluble (data not shown), therefore, biotinylated GN13-E3Q was immobilized to give a relative intensity of 0.2–0.3 nm on streptavidin biosensors. GN13-E3Q was chosen to simplify chemical synthesis of biotinylated cyclic peptides. Binding kinetics of untagged Gαs and Gαq to immobilized GN13-E3Q were quantified using BLI. Association (t = 0-180 s) and dissociation (t = 180-300 s) cycles of Gα proteins were started by dipping sensors into Gα protein solutions and control buffer. Binding signals were double referenced. Mean ± SD, n = 2.

(E) Design of biotinylated GN13-E3Q.

(legend continued on next page)

(F and H) Binding of GD20 (F) or cpGD20 (H) to Gαs, Gα(i/13) and Gαi were quantified using BLI. Biotinylated Gα proteins were immobilized to give a relative intensity of 2–3 nm on streptavidin biosensors, following the same association/dissociation cycles descried in Figure S2C. Binding signals were double referenced and normalized to Gα protein loading and the GD20(or cpGD20)/GDP-bound Gαs binding signal. Mean ± SD, n = 2.

(G and I) Biotinylated GD20 (G) or Biotinylated cpGD20 (I) was immobilized to give a relative intensity of 0.3–0.4 nm on streptavidin biosensors. Binding kinetics of untagged Gαs and Gαq to immobilized GD20 or cpGD20 were quantified using BLI. Association (t = 0-180 s) and dissociation (t = 180-300 s) cycles of Gα proteins were started by dipping sensors into Gα protein solutions and control buffer. Binding signals were double referenced. Mean ± SD, n = 2.

(J) Design of biotinylated GD20 and biotinylated cpGD20 (GD20-F10L).

(K) GD20 inhibited PPI between Gαs/GDP and Gβγ(C68S). GD20 was 100-fold more selective for Gαs than Gαi. Mean ± SD, n = 3.

(L) cpGD20 inhibited PPI between Gαs/GDP and Gβγ(C68S). cpGD20 was nearly 100-fold more selective for Gαs than Gαi. Mean ± SD, n = 3.

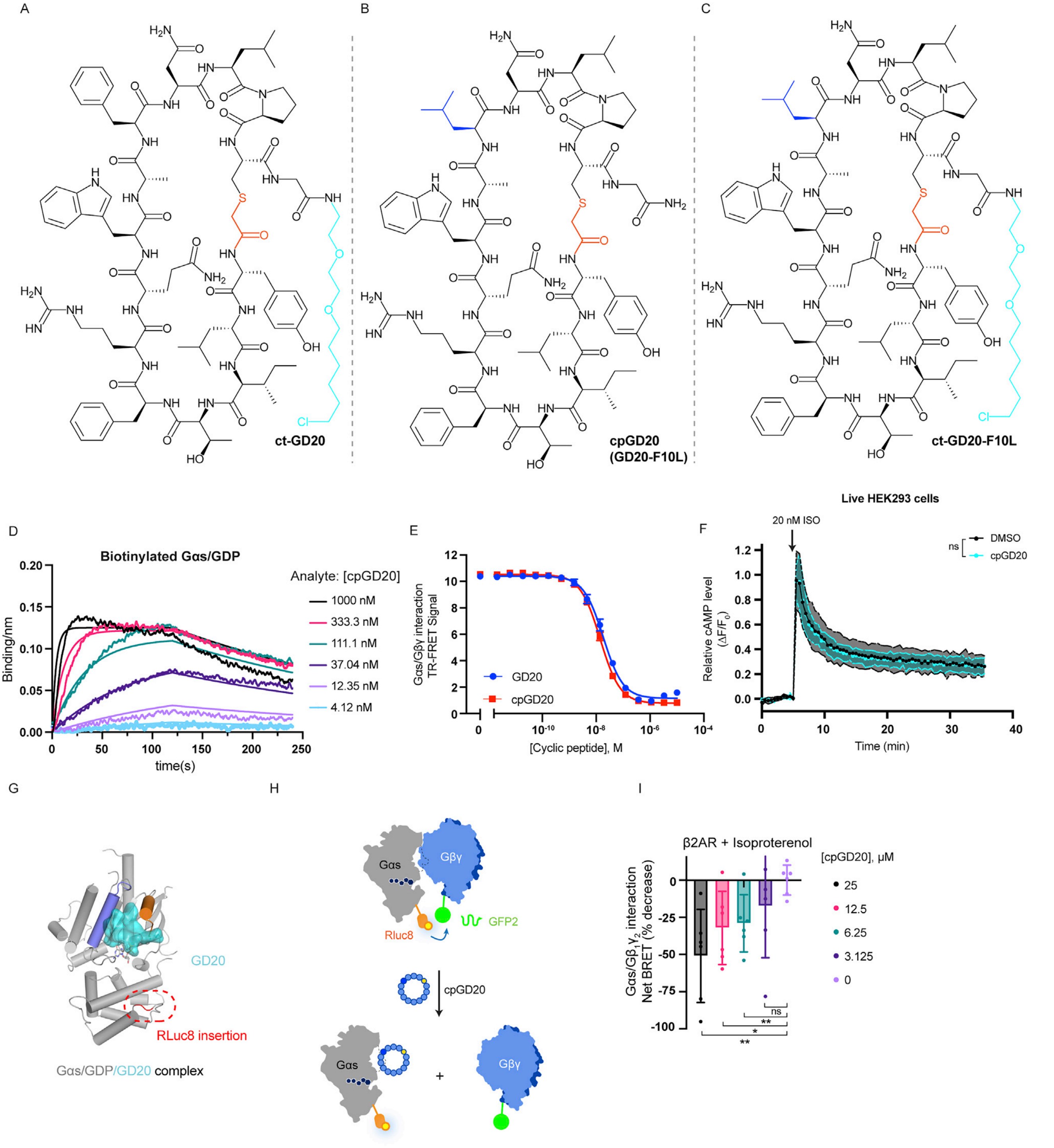

Figure S7. A cell-permeable GD20 analog, cpGD20, is a dual-effect G protein modulator, related to Figure 7

(A–C) Structure of derivatized cyclic peptides. (A) ct-GD20 (B) cpGD20 (GD20-F10L) (C) ct-GD20-F10L.

(D) Binding kinetics of cpGD20 to WT Gαs/GDP were quantified using BLI. Biotinylated WT Gαs/GDP was immobilized to give a relative intensity of 2–3 nm on streptavidin biosensors, following the same association/dissociation cycles descried in Figure S2C. Binding signals were reference-subtracted. The assay was performed in duplicate, and the data represent one of the two replicates.

(E) cpGD20 inhibited PPI between Gαs/GDP and Gβγ(C68S). Mean ± SD, n = 3.

(legend continued on next page)

(F) Pretreament with cpGD20 for 24 h did not inhibited ISO-stimulated cAMP production in live HEK293 cells. Mean ± SD, n = 3. Two-tailed unpaired t tests (data after 5min). ns $p > 0.05$.

(G) The GD20/Gαs complex structure provides structural basis for the Rluc8 insertion. Rluc8 is inserted between αB and αC helices.

(H) Illustration of cpGD20 inhibiting PPI between GαsShort-Rluc and Gβ1/GFP2-γ2 in a BRET2 assay.

(I) cpGD20 inhibited Gαs/Gβγ reassociation in HEK293 cells in a dose dependent manner. Gαs/Gβγ dissociation was measured by BRET2 signal reduction after 1 nM ISO application. BRET signal was normalized to cells that were not treated with ISO and the percentage decrease was calculated based on the net BRET2 signal at [cpGD20] = 0 μM. Mean ± SD, n = 6. Two-tailed unpaired t tests, *$p < 0.05$, **$p < 0.01$, ns $p > 0.05$.
